# Integrative Clustering Reveals a Novel Subtype of Soft Tissue Sarcoma With Poor Prognosis

**DOI:** 10.3389/fgene.2020.00069

**Published:** 2020-02-17

**Authors:** Zhenhua Zhu, Zheng Jin, Haibo Zhang, Mei Zhang, Dahui Sun

**Affiliations:** ^1^ Department of Orthopaedic Trauma, The First Hospital of Jilin University, Changchun, China; ^2^ Department of Immunology, College of Basic Medical Sciences, Jilin University, Changchun, China; ^3^ College of Chemistry, Jilin University, Changchun, China

**Keywords:** soft tissue sarcoma, integration, subtype, consensus clustering, prognosis

## Abstract

**Background:**

Soft tissue sarcomas (STSs) are heterogeneous at the clinical and molecular level and need to be further sub-clustered for treatment and prognosis.

**Materials And Methods:**

STSs were sub-clustered based on RNAseq and miRNAseq data extracted from The Cancer Genome Atlas (TCGA) through the combined process of similarity network fusion (SNF) and consensus clustering (CC). The expression and clinical characteristics of each sub-cluster were analyzed. The genes differentially expressed (lncRNAs, miRNAs, and mRNAs) between the poor prognosis and good prognosis clusters were used to construct a competing endogenous RNA (ceRNA) network. Functional enrichment analysis was conducted and a hub network was extracted from the constructed ceRNA network.

**Results:**

A total of 247 STSs were classified into three optimal sub-clusters, and patients in cluster 2 (C2) had a significantly lower rate of survival. A ceRNA network with 91 nodes and 167 edges was constructed according to the hypothesis of ceRNA. Functional enrichment analysis revealed that the network was mainly associated with organism development functions. Moreover, LncRNA (KCNQ1OT1)-miRNA (has-miR-29c-3p)–mRNA (JARID2, CDK8, DNMT3A, TET1)-competing endogenous gene pairs were identified as hub networks of the ceRNA network, in which each component showed survival significance.

**Conclusion:**

Integrative clustering analysis revealed that the STSs could be clustered into three sub-clusters. The ceRNA network, especially the subnetwork LncRNA (KCNQ1OT1)-miRNA (has-miR-29c-3p)-mRNA (JARID2, CDK8, DNMT3A, TET1) was a promising therapeutic target for the STS sub-cluster associated with a poor prognosis.

## Introduction

Soft tissue sarcomas (STSs) are a set of malignancies that account for 1% of adult cancers (Linch et al., 2014). Approximately 50 STS histological subtypes show heterogeneity in clinical manifestations, histopathological features, and molecular signature (Casali et al., 2018). Despite major breakthroughs in therapies for STSs, standard treatment modalities combined with chemotherapy, radiotherapy, and surgery have failed to improve the overall survival. Although STSs have some common morphologic features and are traditionally aggregated in clinical trials, it is apparent that there are important differences in tumor biology and genetics among these tumors from different anatomic sites (Casali et al., 2018). Consequently, molecular alterations in these heterogeneous tumors should be further explored and new classification systems should be established for the development of more rational, specific, and effective treatments.

Accumulated evidence has shown that transcriptional regulation plays an important role in STS development and progression (Kehlen et al., 2014; Li et al., 2016). The components of the transcriptome, including miRNAs, mRNAs, and lncRNAs, work together and define the biological functions of the tumors. Consensus clustering (CC) (Monti et al., 2003), as an unsupervised subtype discovery method, has been frequently used in many genomic studies (Verhaak et al., 2010; Lehmann et al., 2011). Similarity network fusion (SNF) (Wang et al., 2014) is a computational method that fuses similarity networks for aggregating multi-omics data. The combination of SNF and CC to identify cancer subtypes has achieved more stable and smoother cluster results (Xu et al., 2017).

Competing endogenous RNAs (ceRNAs) are RNAs that indirectly regulate other transcripts by competing for shared miRNAs. Although merely a fraction of the long non-coding RNAs has been functionally characterized, increasing evidence has shown that lncRNAs harboring multiple miRNA response elements (MREs) can act as ceRNAs to sequester miRNA activity and thereby, reduce the inhibition of miRNA on its targets (Salmena et al., 2011). Deregulation of the ceRNA network may lead to human diseases (Barretina et al., 2010). However, to date, little research has been conducted on ceRNA-related lncRNAs associated with the regulatory mechanisms in STS.

The present study aimed to explore the classification of STSs and determine the possible mechanism behind different clusters. The mRNA, lncRNA, and miRNA data of The Cancer Genome Atlas (TCGA) SARC project was used to perform the CC and determine the sub-clusters of STSs. Based on differentially expressed mRNAs, lncRNAs, and miRNAs, a ceRNA network was constructed. The present results demonstrated heterogeneity at the transcriptomic level among STSs. Hence, STSs should be further classified into sub-clusters with biological significance.

## Materials and Methods

### Data Collection

The TCGA database was manually searched and the data transfer tool (provided by GDC Apps) was used to download the level 3 RNASeq, miRNAseq, and somatic mutation data of the specimens, as well as the clinical information of STS patients (https://tcga-data.nci.nih.gov/).

### Consensus Cluster Analysis

The RNAseq (including mRNA and lncRNA) and miRNAseq count data were normalized using the voom function of the limma package (Ritchie et al., 2015). The most variant-expressed 5,000 mRNAs or lncRNAs, defined by median absolute deviation (MAD), and all miRNAs were used for CC. The samples were clustered through the “ExecuteSNF.CC” function of the R package “Cancer Subtypes” (Xu et al., 2017). This function is a combined process of SNF (Wang et al., 2014) and CC (Gaujoux and Seoighe, 2010) for cancer subtype identification. First, the SNF separately calculates the similarity between patients using RNAseq data and miRNA data. Then, the similarities between patients from these two data types are integrated by a cross-network diffusion process to construct a fusion patient similarity matrix that is used as the sample distance for CC. After the samples are allocated into clusters, the silhouette width (Rousseeuw, 1987) is determined to evaluate the clustering results. This combines the factors of cohesion and separation.

### Cluster Characteristic Identification

To compare our sub-clusters with histological subtypes, the distribution of the histological subtypes in each cluster was determined. An overall survival analysis was also conducted within the three clusters using the R package survminer. The mutation rates of tumor protein p53 (TP53) and RB transcriptional corepressor 1 (RB1), which are the two most mutated tumor suppressor genes in STS (Barretina et al., 2010), were also measured and compared between the clusters using chi-squared tests. The sub-cluster specific genes were screened by a two-step method. In the first step, the differential genes of one sub-cluster and the other two sub-clusters were calculated using the limma package (Smyth, 2005), with a threshold of |log2 (fold-change [FC])| > 2.0 and a *P*-value < 0.01. The second step was to take the intersection of the two parts of the differential genes. These were the sub-cluster specific genes.

### CeRNA Network Construction

In this study, sub-clusters were identified by the expression of lncRNAs, miRNAs, and mRNAs and their internal regulatory relationships were observed between the genes according to the hypothesis of ceRNA. R package GDCRNATools (Xu et al., 2017), which utilized the target information of miRNA–mRNA and miRNA–mRNA of Starbase V2.0 (Li et al., 2014), was utilized to construct the ceRNA network. The target information in Starbase 2.0 was predicted by the miRanda prediction program (Betel et al., 2008) and validated in the CLIP-seq data of the AGO protein (a major member of the RISC complex). Specifically, the “gdcCEAnalysis” function of GDCRNATools was used to identify the competing lncRNA–mRNA pairs: (a) a lncRNA–mRNA pair should have an overlap of miRNA targets (based on the Starbase lncrna–mirna and mrna–mirna prediction data), (b) the significant positive correlation between lncRNA and mRNA expression data should be verified by Pearson’s correlation (cutoff *P*-value < 0.05), and (c) the expression of both lncRNA and mRNA showed be negatively correlated with the expression of shared miRNAs (cutoff *P*-value < 0.05). In the above process, competing gene pairs are connected through miRNAs, forming a ceRNA regulatory network. To obtain insight into the function of the ceRNA network, gene ontology (GO) enrichment analysis was conducted using the STRING database (v11.0) (Szklarczyk et al., 2019).

### Identification and Survival Analysis of the Subnetwork

Cox regulation analysis and KM plot analysis were conducted on each node of the network. Moreover, if all the components of a competing gene pair (lncRNA–miRNA–mRNA) showed survival significance (Cox *P*-value < 0.05 and logRank *P*-value < 0.05), the pair was selected as part of the hub ceRNA network.

For KM survival analysis of the subnetwork, the samples were divided into two groups according to the median expression level of the nodes. For all the mRNAs of the subnetwork, the samples were divided according to the first principal of the principal components analysis (PCA) of all the mRNAs. To explore the survival significance of the whole subnetwork (Wang et al., 2019), a Cox regulation model was utilized using all the nodes of the subnetwork as variables and the risk score of each sample was calculated. Then, the samples were divided into two groups by the median risk score.

## Results

### Clinical Details of Samples

The TCGA-SARC project contained the RNAseq, miRNAseq, and clinical information of 247 soft sarcoma specimens. These specimens were obtained from 113 males and 134 female patients with a median age of 61 years (range: 21 – 90 years old). The histologic subtypes included 99 leiomyosarcomas (LMSs), 56 dedifferentiated liposarcomas, 28 pleomorphic malignant fibrous histiocytomas (MFHs), 22 myxofibrosarcomas, 20 undifferentiated pleomorphic sarcomas (UPSs), and 22 other types of STSs. The detailed histological subgroup distribution is presented in **Table 1**.

**Table 1:** Clinical statistical information of patients in the training group.

Characteristics	Detail
Histological type	
Dedifferentiated liposarcoma	56
Leiomyosarcoma (LMS)	99
Myxofibrosarcoma	22
Pleomorphic malignant fibrous histiocytoma (MFH)/undifferentiated pleomorphic sarcoma	28
Undifferentiated pleomorphic sarcoma (UPS)	20
Others	22
Age	61 (21–90)
Gender	
Male	113
Female	134
Metastasis	
Yes	55
No	110
Not available	82
Tumor depth	
Deep	176
Superficial	19
Not available	52
Necrosis	
0% (no necrosis or no mention of necrosis)	68
< 10% (focal necrosis)	35
Moderate necrosis (> = 10, < 50%)	56
Extensive necrosis (> 50%)	11
Not available	77
Race	
Asian	6
White	216
Black or African American	18
Not evaluated	7

### STSs Could Be Classed Into Three Optimal Sub-Clusters

**Figure 1:**
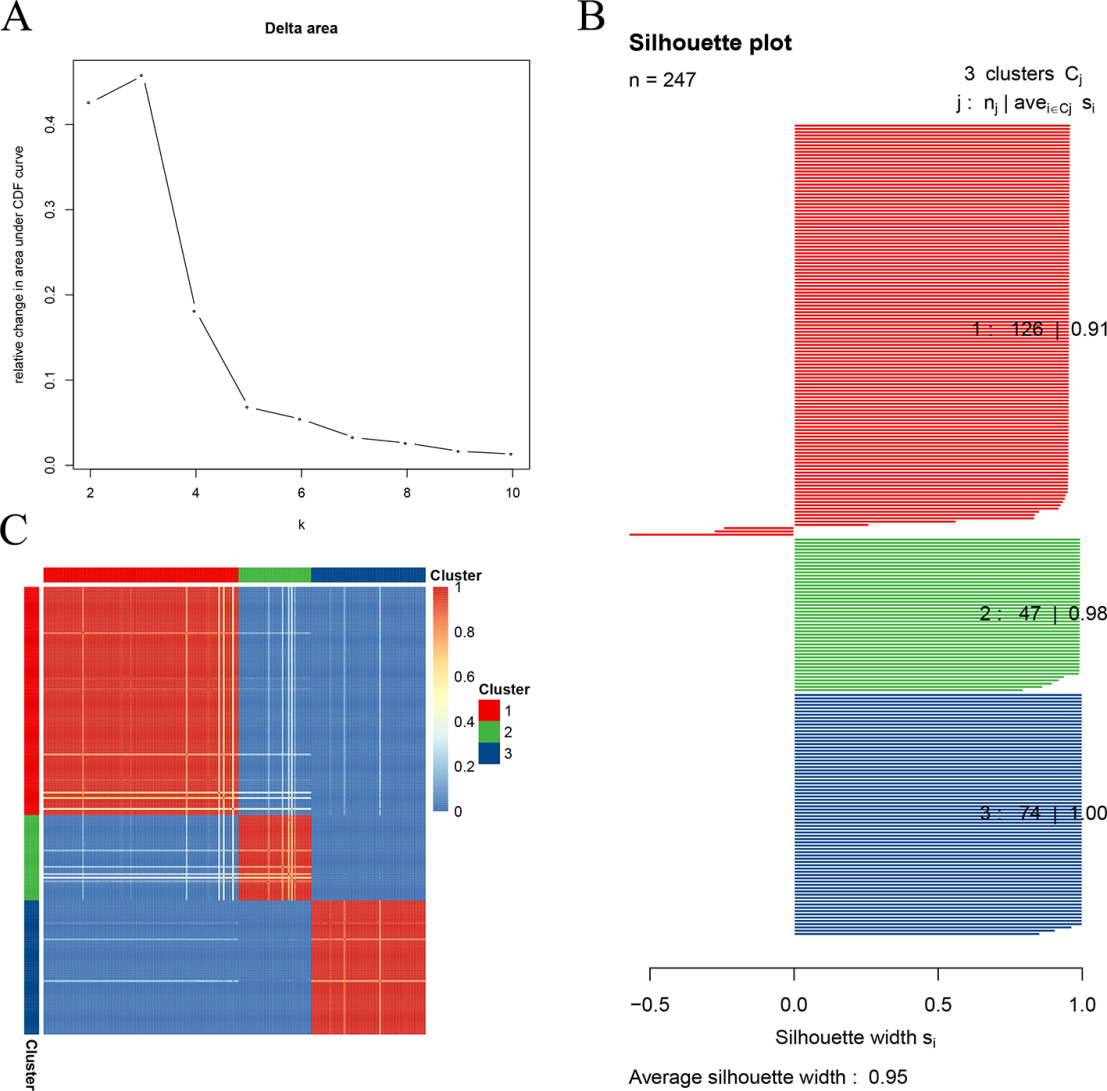
Clustering soft tissue sarcomas (STSs) in the training test dataset. **(A)** Relative change in the area under the empirical cumulative distribution (CDF) curve. **(B)** The specimens in the subgroup are well-allocated to their group, rather than to the other groups (average silhouette width: 0.95). **(C)** Sample distance in the training set. The darker the color, the closer the samples became, and the more similar the samples were.

The optimal number of clusters was estimated by calculating the relative change in the area under the empirical cumulative distribution (CDF) curve, which reflected the consensus distribution (Monti et al., 2003). The biggest change was observed when the cluster number (k) was set to 3 (**Figure 1A**), so the STSs were sub-clustered into three clusters. As for the silhouette width, the silhouette width values vary between −1 and 1. The more it approaches 1, the better the cohesion and separation degree becomes. The average silhouette width of all points is the total silhouette width of the clustering results. This technique provides a succinct graphical representation of how well each object lies within its cluster. In this study, the width of each cluster and the total width were close to 1, indicating a higher similarity among the members within the cluster compared to the members in different clusters (**Figure 1B**). The sample distance was visualized in heatmap and showed that the samples within the cluster were close to each other, whereas the samples between the clusters were far from each other (**Figure 1C**).

### Characteristics of the Sub-Clusters

**Figure 2:**
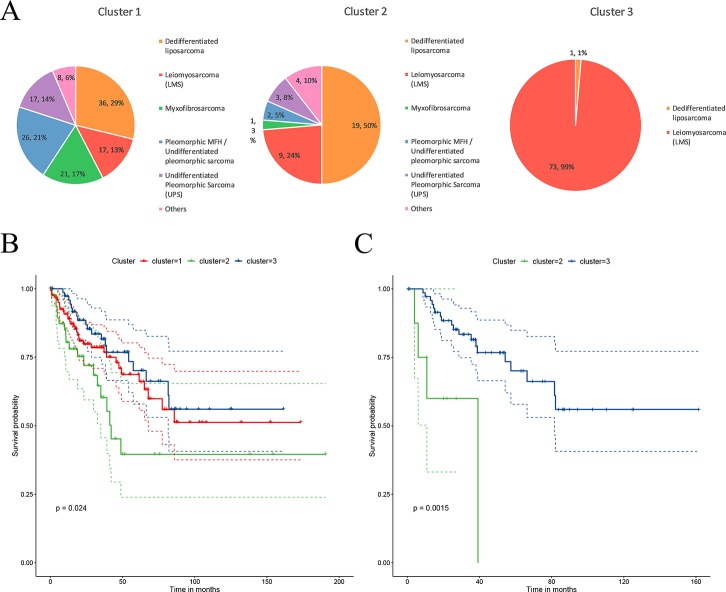
Characteristics of the sub-clusters. **(A)** The distribution of tumor histological types in each sub-cluster. **(B)** Patients in the three sub-clusters had significant differences in survival. **(C)** The leiomyosarcoma (LMS) patients in C2 had a significantly lower overall survival.

The STSs were clustered into three sub-clusters through CC. The distribution of the patients in each cluster is presented in **Figure 2A**. C1 contains various histological types; C2 mainly contains two types of histological types, dedifferentiated liposarcoma and LMS (74%); and C3 mainly contains LMS (99%). Dedifferentiated liposarcoma comprised a high proportion of both the C1 and C2 clusters. LMS was mainly present in C3 and had a certain distribution in C1 and C2. Myxofibrosarcoma, pleomorphic MFH/UPS, and UPS were mainly present in C1. The survival analysis revealed that patients in cluster 2 (C2) had a significantly lower rate of survival (*P* = 0.02) compared to those in cluster 1 (C1) and cluster 3 (C3) (**Figure 2B**). Because the main histological type in C3 was LMS, to verify that the difference in survival was caused by molecular sub-clusters rather than histological types, survival analysis for the LMSs in the C2 and C3 clusters was conducted. The overall survival rate of LMS patients in C2 was significantly (*P =* 0.0015) lower than that in the patients in C3 (**Figure 2C**). The sub-cluster information for each sample is provided in **Supplementary Table 1**.

**Figure 3:**
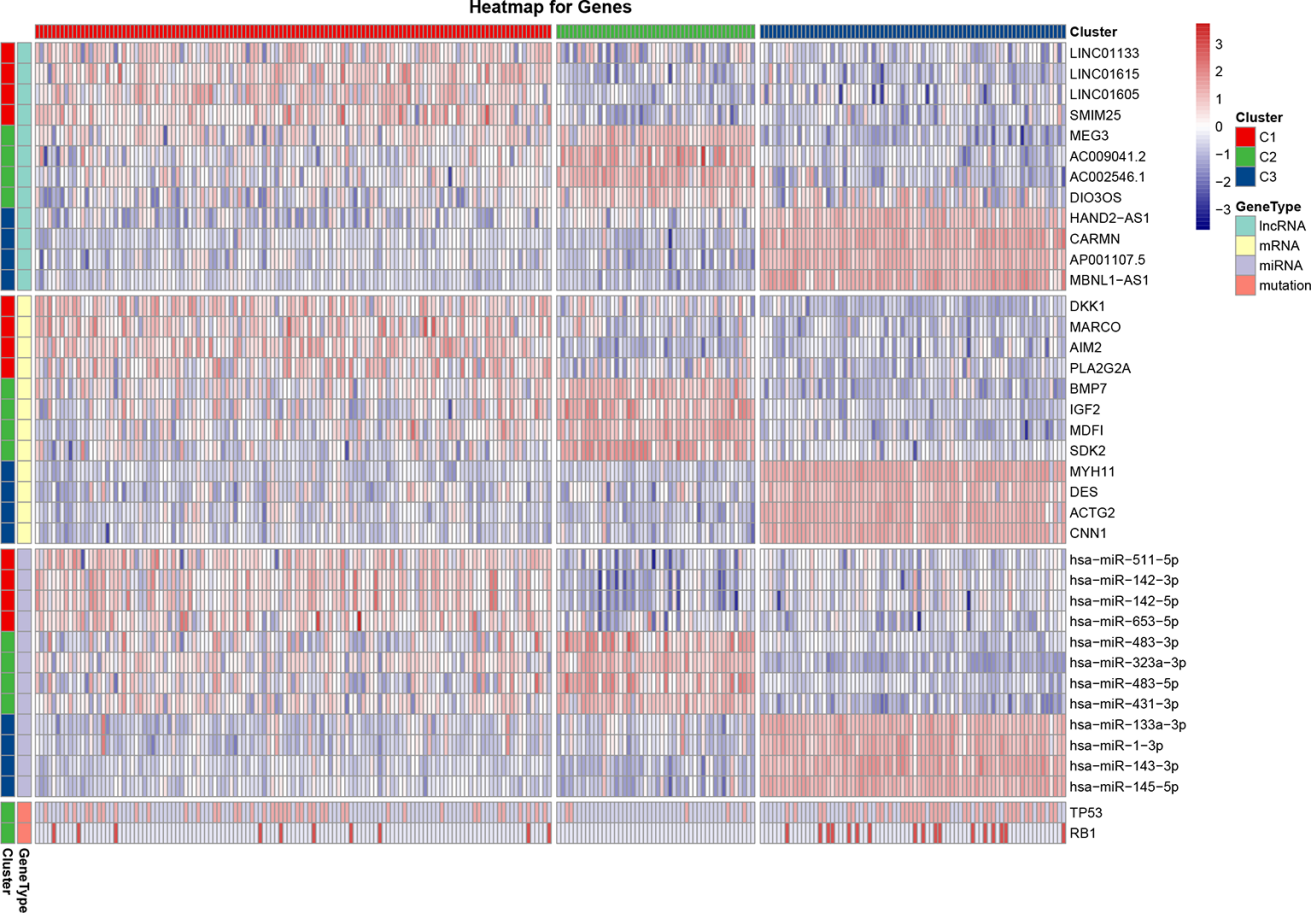
Heatmap of the most differentially expressed lncRNAs, mRNAs, miRNAs, and variant mutation genes of each sub-cluster. The expression Z-score data were normalized. The red color indicates high expression, the blue color indicates low expression, and a darker color indicates a greater difference from the mean.

For each cluster, a differential analysis was conducted twice for the other two clusters. Genes with significantly high expression in both differential analyses were defined as sub-cluster-specific genes. The genes with the greatest sum of FCs were defined as marker genes of the sub-clusters, as shown on the heatmap (**Figure 3**). As for marker genes, LncRNA LINC01133, mRNA dickkopf WNT signaling pathway inhibitor 1 (DKK1), and miRNA has-miR-511-5p were the marker genes of C1; LncRNA maternally expressed 3 (MEG3), mRNA bone morphogenetic protein 7 (BMP7), and miRNA has-miR-483-3p were the marker genes of C2; and LncRNA HAND2-AS1, mRNA myosin heavy chain 11 (MYH11), and miRNA has-miR-133a-3p were the marker genes of C3. As the most commonly mutated genes in the STSs, TP53 and RB1 had significantly (*P* < 0.05) higher mutation rates in the C1 (TP53: 17.0%, RB1: 4.0%) and C3 clusters (TP53: 15.0%, RB1: 6.9%) than in the C2 cluster (TP53: 2.0%, RB1: 0.0%) (**Figure 3**). The mutation rate of the genes for each sub-cluster is provided in **Supplementary Table 2**.

### The Components of the CeRNA Network and Functional Enrichment Results

**Figure 4:**
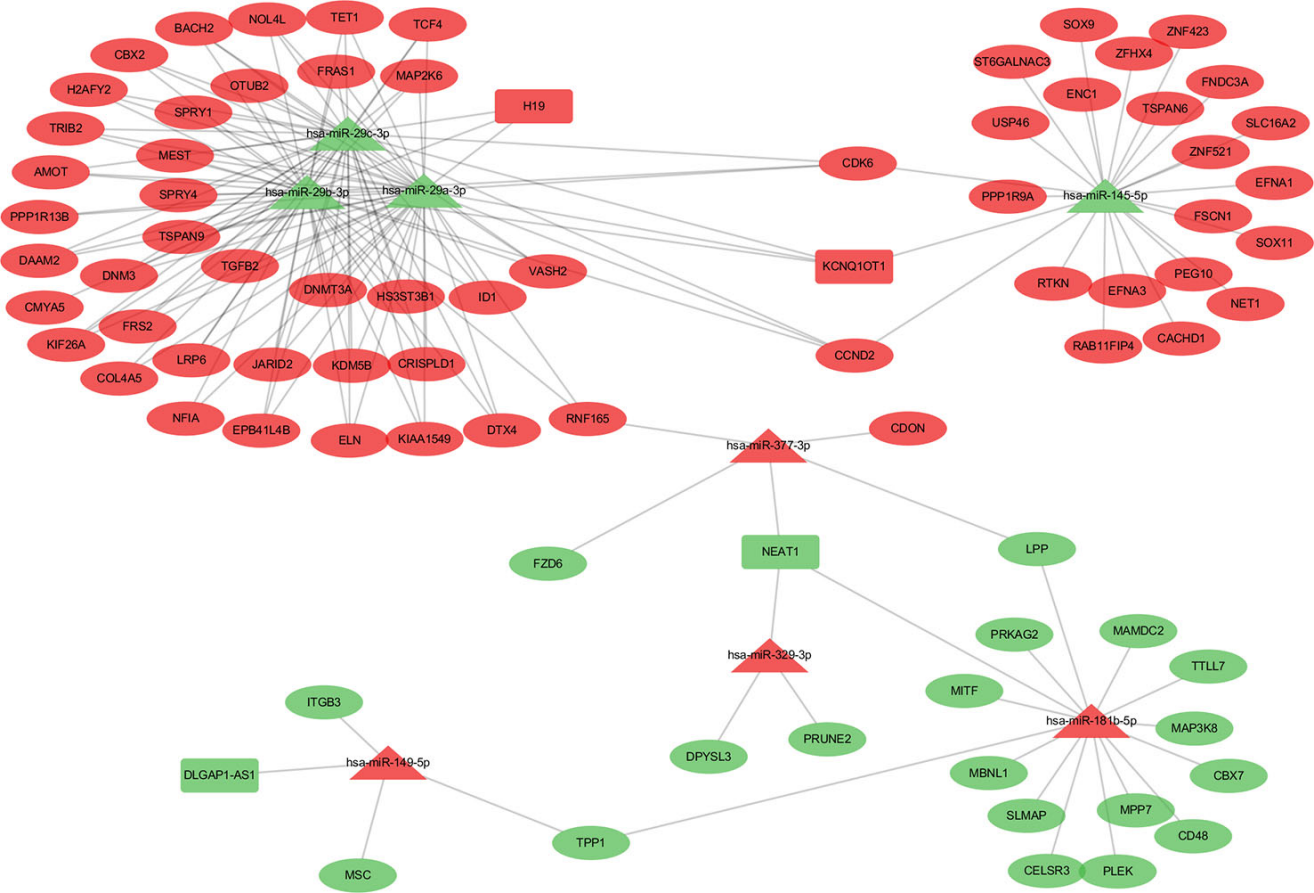
The competing endogenous RNA (ceRNA) network constructed from differentially expressed genes. The different shapes represent different gene types: the rectangle is lncRNA, the triangle is miRNA, and the ellipse is mRNA. Red color indicates a high expression in C2 and green indicates a low expression compared to the C1&C3 clusters.

**Figure 5:**
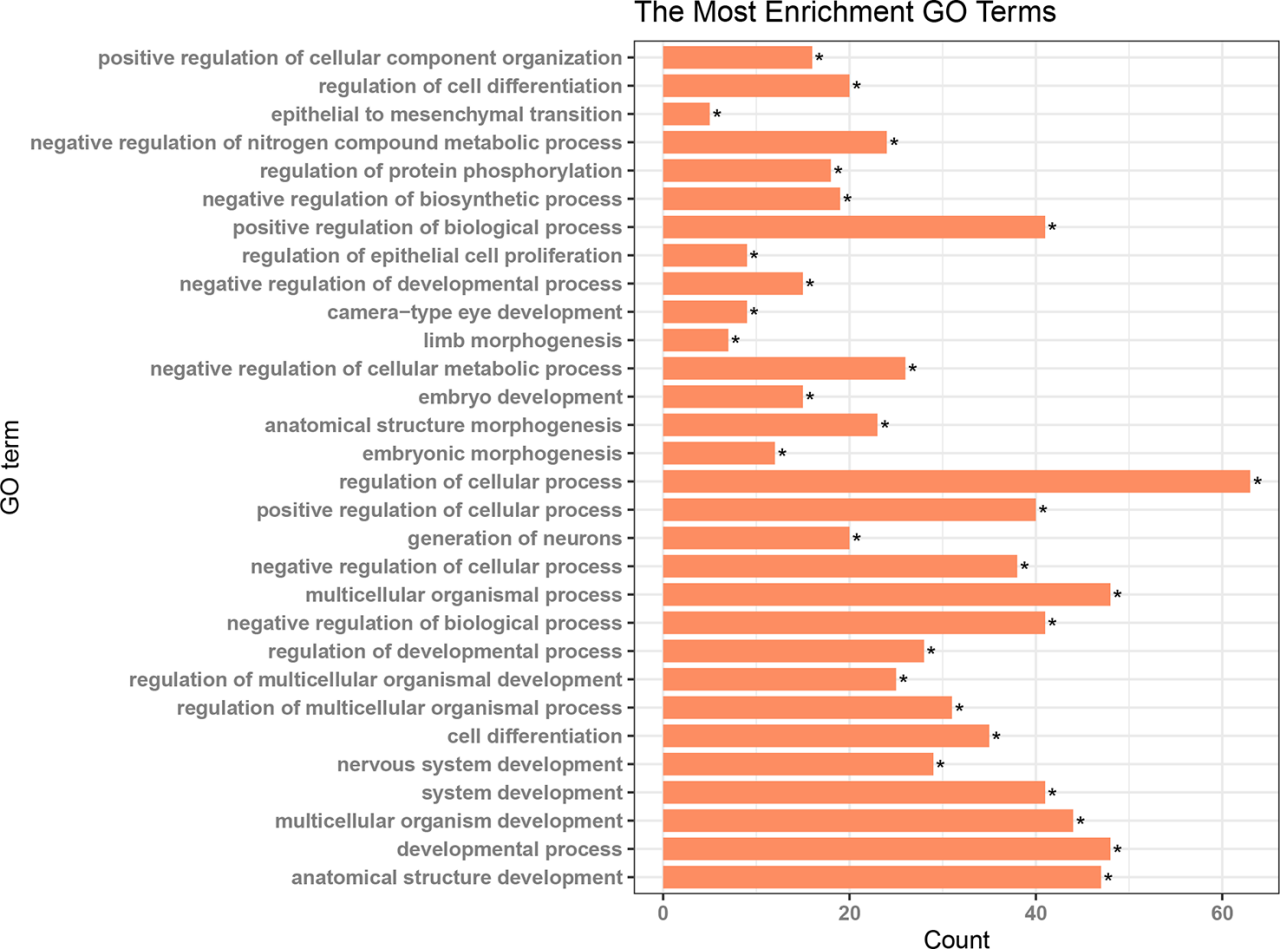
Results of functional enrichment analysis for the nodes in the ceRNA network *p < 0.05.

Patients in the C2 cluster showed significantly worse prognoses that were not driven by mutations of the tumor suppressor genes TP53 and RB1. A ceRNA network constructed based on the differentially expressed genes (lncRNAs, miRNAs, and mRNAs) between C2 and C1&C3, may help us understand the biological characteristics of C2. In the differential analysis of C2 and C1&C3, 100 differentially expressed LncRNAs, 152 differentially expressed miRNAs, and 1,663 differentially expressed mRNAs were identified. Under the ceRNA hypothesis, a ceRNA network with 91 nodes (4 lncRNAs, 8 miRNAs, and 79 mRNAs, **Supplementary Table 3**) and 167 edges was **Figure 4**. In the network, the expression of miRNAs was negatively correlated with the expression of their lncRNA and mRNA targets, whereas and the expression of lncRNAs and mRNAs was positively correlated. Competing gene pairs were connected by shared miRNAs, forming a single network. Functional enrichment analysis indicated that ceRNA mainly functioned in development and cellular processes (**Figure 5**, **Supplementary Table 4**).

### Hsa-miR-29c-3p Centered Sub-ceRNA Network Showed Survival Significance

**Figure 6:**
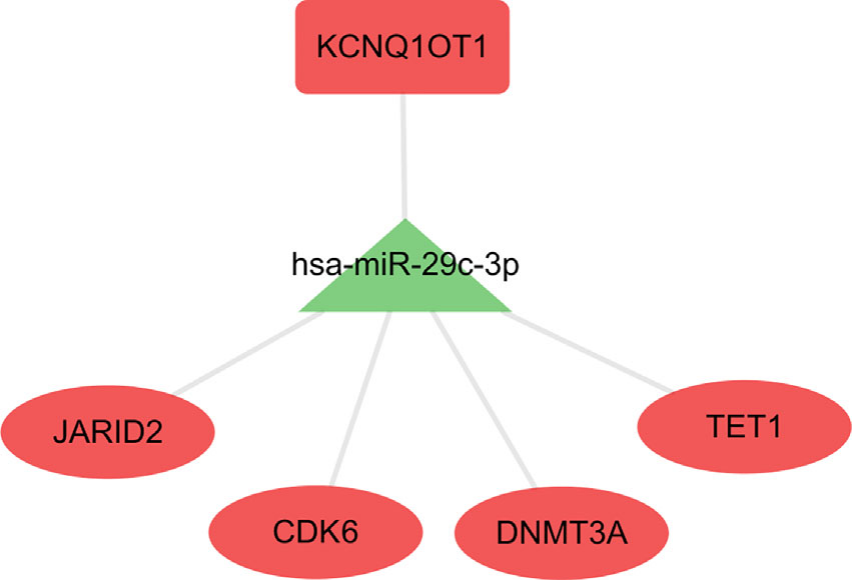
The sub-ceRNA network, in which each node showed survival significance.

**Figure 7:**
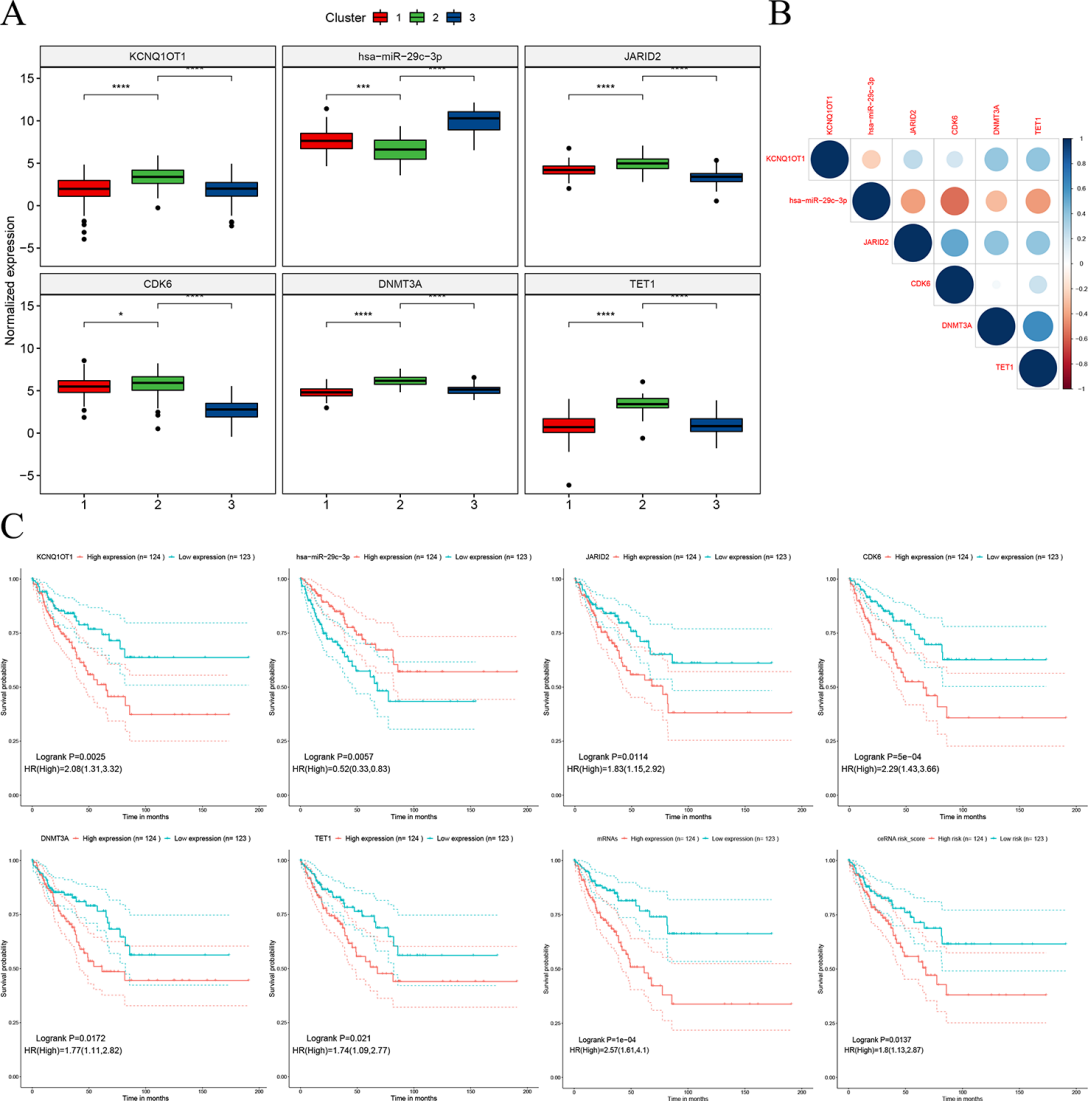
Characteristics of the sub-ceRNA network. **(A)** Expression of nodes in the sub-clusters. **(B)** Expression correlation between the nodes. **(C)** Survival significance of the nodes and the sub-ceRNA network *p < 0.05; ***p < 0.001; ****p < 0.0001.

The hub network was identified by the extracted competing gene pairs, each component of which showed survival significance (**Supplementary Table 3**). In the subnetwork, LncRNA KCNQ1OT1 (KCNQ1 opposite strand/antisense transcript 1) and JARID2 (jumonji and AT-rich interaction domain containing 2), CDK6 (cyclin-dependent kinase 6), DNMT3A (DNA methyltransferase 3 alpha), and TET1 (tet methylcytosine dioxygenase 1) competitively bound hsa-miR-29c-3p (**Figure 6**). KCNQ1OT1 and mRNAs showed significantly higher expression levels in the C2 cluster compared to the C1&C3 clusters (**Figure 7A**). KCNQ1OT1 was negatively correlated with the expression of has-miR-29c-3p and positively correlated the expression of mRNAs. All the mRNAs were negatively correlated with the expression of hsa-miR-29c-3p (**Figure 7B**). As for survival significance, high expression of KCNQ1OT1 and mRNAs was associated with worse patient prognosis, whereas high expression of hsa-miR-39c-3p indicated a better prognosis. As a representative of all the mRNAs, higher expression of the first principle of mRNAs indicated a poor prognosis. The risk score calculated from Cox regulation was used to evaluate the survival significance of the whole network. A higher risk score indicated a poorer overall survival (**Figure 7C**).

## Discussion

STSs form a rare group of malignancies differing in biology, behavior, and sensitivity to treatment (Sleijfer and Gelderblom, 2014). Although there are up to 50 histological subtypes, treatment for each subtype remains similar. For *in situ* STSs, surgical resection is the main method, supplemented with radiotherapy (Mehren et al., 2018). For most metastatic STSs, doxorubicin ± ifosfamide is the standard first-line therapy (Ratan and Patel, 2016; Mehren et al., 2018). Unfortunately, patients have a 10%–30% recurrence rate and a 30%–40% distant metastasis rate, even after initial treatment (Frezza et al., 2017). Molecular targeted therapy, targeting tumor-specific genomic changes, can achieve maximum efficacy while minimizing side effects, bringing options to the treatment of STSs. Currently, several drugs targeting vascular endothelial growth factor (VEGF) for STS have entered the clinical trial stage and some of them have shown good clinical effects (Kuenen et al., 2003; Suttle et al., 2004; D’Adamo et al., 2005; Dalal et al., 2005). This is a good start, but because STSs are rare and there are too many histological subtypes, clinical research for STSs is challenging. Most clinical studies are based on one or several specific histological types, so clinical trials are progressing slowly due to the limited number of patients (Sleijfer and Gelderblom, 2014). Genotyping methods based on genomics can classify tumors with similar genomic characteristics into the same category, thus facilitating the screening of molecular targets and the conduct of clinical trials.

Transcriptome regulation is important in the performance of cellular biological functions. CC analysis based on transcriptome data clearly divided the STSs into three sub-clusters, suggesting that the STSs had similar characteristics within the clusters and different characteristics between the clusters at the transcriptome level. Moreover, significant differences in survival were found between the clusters. The prognosis of patients in the C2 cluster was worse and based on a separate survival analysis of the LMS samples in C2 and C3 clusters. We believe that the differences are due to molecular subtypes rather than histological types **Supplementary Figure 1**. This has also been verified in previous studies on LMS. LMS is significantly different at the genetic level and can be classified into subgroups (Beck et al., 2010; Guo et al., 2015), showing different responses to treatment (Guo et al., 2015).

Tumor-suppressor gene mutation is an important pathogenic mode of tumors. TP53 and RB1 are the most common tumor suppressor gene mutations in STS and both of these mutations cause abnormalities in the cell cycle that lead to cancer (Brohl et al., 2015). In the poor prognosis cluster C2, a significantly lower mutation rate in TP53 and RB1 was observed compared to the C1&C3 clusters. Koehler et al. (2016) reported that TP53 mutations indicated a better response to the VEGF target drug pazopanib and better PFS. The possible mechanism is that wild-type TP53 suppresses angiogenesis by the transcriptional suppression of VEGF expression. The loss of function of TP53 produced significantly more VEGF (Zhang et al., 2000). Moreover, the TP53-VEGF signaling pathway seems to be dependent on the function of RB1 (Farhang Ghahremani et al., 2013). Thus, a loss of function mutation in either TP53 or RB1 will directly contribute to tumor angiogenesis and the potential sensitivity to anti-angiogenic therapy (Koehler et al., 2016). Based on these studies, we hypothesized that anti-angiogenic drugs used in patients in the C1&C3 group might achieve better efficacy than in the C2 group.

CeRNA is a kind of post-transcriptional regulation whereby transcriptions cross-regulate each other by competing for shared miRNAs (Salmena et al., 2011). It may explain disease processes and present opportunities for new therapies (Salmena et al., 2011). In this study, we used the hypothesis of ceRNA to construct a ceRNA network based on the differential genes between C2 and C1&C3, hoping to partially explain the biological characteristics of C2 and provide some potential therapeutic targets for C2. The results of the enrichment analysis of the network nodes indicated that this ceRNA was mainly related to organism development functions. Differences in the tumor tissue progression direction may contribute to differences in overall survival and need to be further analyzed. Moreover, more attention showed to be focused on the sub-networks KCNQ1OT1/hsa-miR-29c-3p/JARID2, CDK6, DNMT3A, and TET1 extracted from the whole network, where each node showed survival significance. The KCNQ1OT1 transcript is the antisense to the KCNQ1 gene and is an unspliced long non-coding RNA. It interacts with chromatin and regulates the transcription of multiple target genes through epigenetic modifications. It was reported to have a role in the regulation of proliferation and cisplatin in tongue cancer (Zhang et al., 2018) and colorectal carcinogenesis (Zhang et al., 2019). Furthermore, the knockdown of KCNQ1OT1 depressed chemoresistance to paclitaxel in lung adenocarcinoma (Ren et al., 2017). Hsa-miR-29c-3p is known as a tumor suppressor gene. The overexpression of hsa-miR-29c-3p reduced cell proliferation and migration in colorectal cancer (Zhang et al., 2017), increased the chemosensitivity of pancreatic cancer cells (Huang et al., 2018), and reduced the cisplatin resistance of non-small cell lung cancer cells (Sun et al., 2018). JARID2 encodes a Jumonji and AT-rich interaction domain (ARID)-domain-containing protein. The encoded protein is a DNA-binding protein that functions as a transcriptional repressor. It was reported to promote the invasion and metastasis of hepatocellular carcinoma by facilitating epithelial-mesenchymal transition through the PTEN/AKT signaling pathways (Lei et al., 2016) and is essential for the maintenance of tumor-initiating cells in bladder cancer (Zhu et al., 2017). The protein encoded by CDK6 is a member of the CMGC family of serine/threonine protein kinases. This kinase is a catalytic subunit of the protein kinase complex that is important for cell cycle G1 phase progression and G1/S transition. CDK6 serves as the target of multiple miRNAs and had demonstrated important roles in tumor growth (Agirre et al., 2009; Lu et al., 2017; Yuan et al., 2017). DNMT3A encodes a DNA methyltransferase that is thought to function in *de novo* methylation, rather than maintenance methylation, which impacts the expression of p21 and plays a role in determining doxorubicin-induced senescence and apoptosis in HCT116 colorectal cancer cells (Zhang et al., 2011). TET1 encodes a demethylase that belongs to the TET (ten-eleven translocation) family. Members of the TET protein family play a role in the DNA methylation process and gene activation. High expression of TET1 indicates a poor prognosis in cytogenetically normal acute myeloid leukemia (Wang et al., 2018) and promotes cisplatin-resistance in ovarian cancer (Han et al., 2017). As for the competing pairs, miR-29b/c was shown to suppress the downstream gene DNMT3A, and in turn, miR-29b/c was suppressed by DNMT3A in a DNA methylation-dependent manner in gastric cancer (He et al., 2015). In our study, based on the ceRNA hypothesis, tumor-promoting genes, including KCNQ1OT1, JARID2, CDK6, DNMT3A, and TET1, competitively bound the tumor suppressor gene hsa-29c-3p. In that case, targeting nodes of the sub-ceRNA network may also affect the other nodes in the sub-network, inducing corresponding changes in the biological processes. Therefore, this sub-ceRNA network can provide new ideas for C2 treatment.

In conclusion, integrative clustering analysis revealed that STSs could be clustered into three sub-clusters. The ceRNA network, especially the subnetwork LncRNA (KCNQ1OT1)-miRNA (has-miR-29c-3p)–mRNA (JARID2, CDK8, DNMT3A, TET1) represents a promising therapeutic target for treating the STS sub-cluster of patients with a poor prognosis.

## Data Availability Statement

All datasets generated for this study are included in the article/**Supplementary Material**.

## Author Contributions

ZZ and DS designed the study. ZZ and ZJ performed the data collection. HZ performed the data analysis. ZZ and MZ drafted the manuscript. All authors read and approved the final version of the manuscript.

## Funding

This study was supported by the Special Projects of Health in Jilin Province (3D5148273428) and the Key R&D Program of Guangdong Province (2018B090906001).

## Conflict of Interest

The authors declare that the research was conducted in the absence of any commercial or financial relationships that could be construed as a potential conflict of interest.

## Supplementary Material

Supplementary Figure 1 The overall survival analysis of different histological types of STSs in the training dataset.
