# Supplementary figures and images for: Integrative Clustering Reveals a Novel Subtype of Soft Tissue Sarcoma With Poor Prognosis
Source: Front Genet. 2020 Feb 17;11:69. doi: 10.3389/fgene.2020.00069 (PMC7038822; doi:10.3389/fgene.2020.00069)

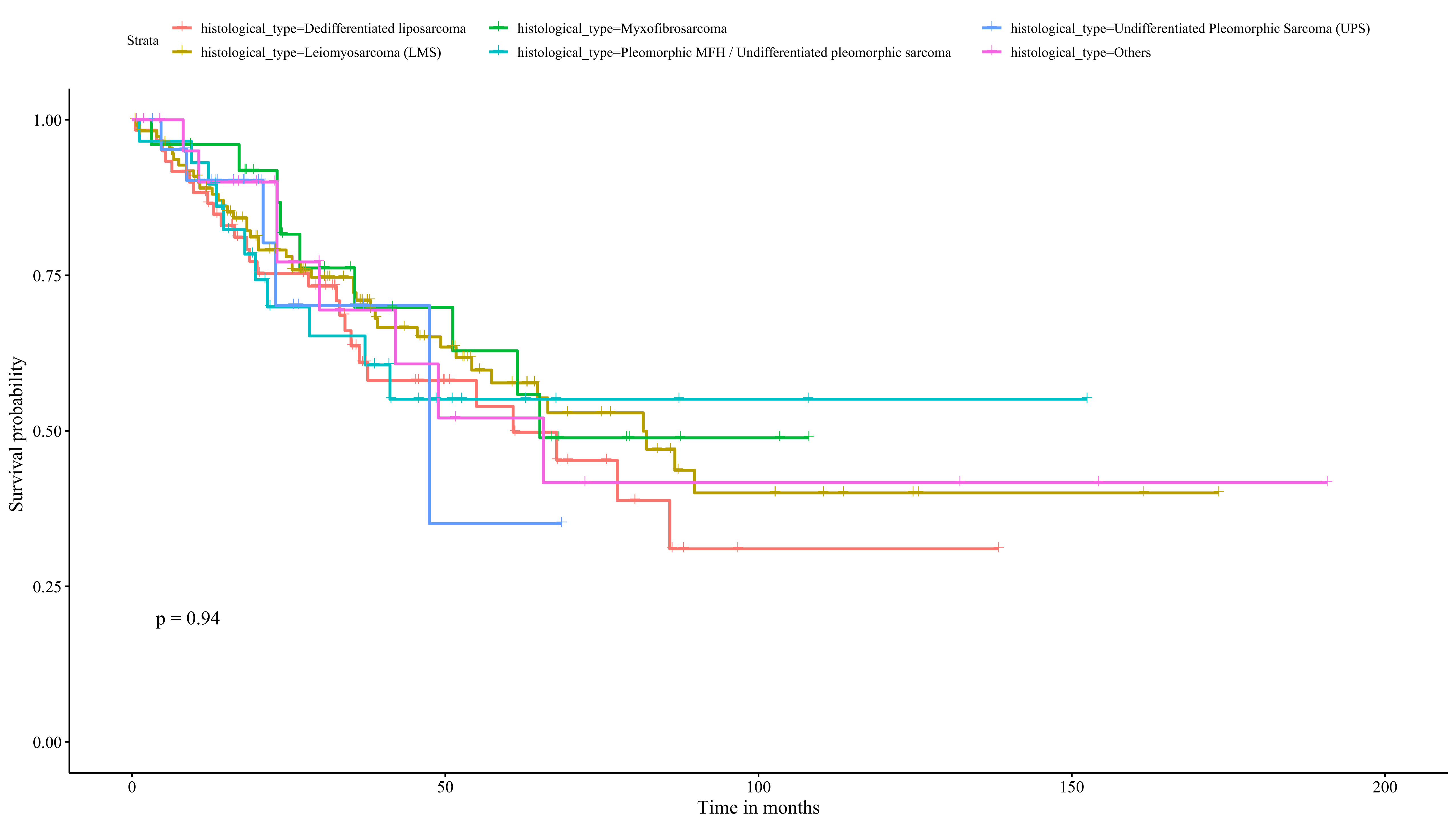

Supplement: Supplementary Figure 1 — The overall survival analysis of different histological types of STSs in the training dataset. [file Image_1.tiff]
