# Supplementary material for: Integrative Clustering Reveals a Novel Subtype of Soft Tissue Sarcoma With Poor Prognosis
Source: Front Genet. 2020 Feb 17;11:69. doi: 10.3389/fgene.2020.00069 (PMC7038822; doi:10.3389/fgene.2020.00069)
Supplement: Supplementary file 2 [file Table_1.docx]

Supplementary Table 1: Sub-cluster information for samples

| Samples | Gender | Age | Race | Event | Days until last follow up | Cluster |
| --- | --- | --- | --- | --- | --- | --- |
| TCGA-UE-A6QU | FEMALE | 90.1 | ASIAN | Alive | 0 | 1 |
| TCGA-RN-AAAQ | MALE | 52.4 | WHITE | Alive | 510 | 1 |
| TCGA-FX-A2QS | FEMALE | 61.4 | WHITE | Alive | 1495 | 2 |
| TCGA-MJ-A850 | MALE | 28.6 | WHITE | Alive | 681 | 2 |
| TCGA-DX-AB2X | FEMALE | 73.2 | WHITE | Dead | 404 | 1 |
| TCGA-DX-A8BN | FEMALE | 78.8 | WHITE | Alive | 1161 | 1 |
| TCGA-HB-A43Z | FEMALE | 58.2 | WHITE | Alive | 1126 | 3 |
| TCGA-DX-A8BS | FEMALE | 68.6 | BLACK OR AFRICAN AMERICAN | Dead | 850 | 1 |
| TCGA-DX-A6YX | FEMALE | 68.1 | Unknown | Dead | 513 | 1 |
| TCGA-DX-A3LY | MALE | 66.2 | WHITE | Alive | 1017 | 1 |
| TCGA-DX-A8BL | MALE | 59.5 | WHITE | Alive | 1373 | 1 |
| TCGA-MB-A5YA | MALE | 63.3 | WHITE | Alive | 15 | 3 |
| TCGA-3R-A8YX | MALE | 66.9 | Not Evaluated | Dead | 17 | 1 |
| TCGA-DX-A8BH | MALE | 87 | WHITE | Dead | 439 | 1 |
| TCGA-IW-A3M6 | FEMALE | 59.4 | ASIAN | Alive | 688 | 3 |
| TCGA-DX-A3U9 | MALE | 85 | WHITE | Dead | 456 | 3 |
| TCGA-HB-A3L4 | FEMALE | 46.7 | WHITE | Dead | 1061 | 3 |
| TCGA-K1-A6RU | FEMALE | 66.7 | WHITE | Dead | 711 | 1 |
| TCGA-MB-A8JL | FEMALE | 53.5 | WHITE | Alive | 600 | 2 |
| TCGA-RN-A68Q | FEMALE | 63 | WHITE | Alive | 22 | 1 |
| TCGA-LI-A9QH | FEMALE | 72.5 | WHITE | Dead | 540 | 1 |
| TCGA-3B-A9HT | MALE | 53.8 | WHITE | Alive | 1665 | 1 |
| TCGA-3B-A9HR | FEMALE | 38.5 | BLACK OR AFRICAN AMERICAN | Dead | 2694 | 3 |
| TCGA-3B-A9HO | MALE | 75.8 | WHITE | Alive | 959 | 1 |
| TCGA-DX-A6YU | FEMALE | 50.3 | WHITE | Alive | 1045 | 1 |
| TCGA-DX-AB30 | FEMALE | 54 | WHITE | Dead | 695 | 2 |
| TCGA-K1-A6RT | MALE | 48.6 | WHITE | Alive | 528 | 1 |
| TCGA-DX-A3LT | MALE | 62.6 | WHITE | Alive | 1492 | 1 |
| TCGA-3B-A9I3 | MALE | 63 | WHITE | Alive | 808 | 3 |
| TCGA-DX-A6BE | MALE | 77.7 | WHITE | Alive | 881 | 1 |
| TCGA-DX-A3U7 | MALE | 67.7 | WHITE | Dead | 1552 | 3 |
| TCGA-HS-A5N8 | FEMALE | 70 | Not Evaluated | Dead | 22 | 2 |
| TCGA-WK-A8Y0 | FEMALE | 50 | WHITE | Dead | 688 | 1 |
| TCGA-DX-A6BB | MALE | 74.4 | WHITE | Alive | 1105 | 3 |
| TCGA-IS-A3K8 | FEMALE | 44.6 | WHITE | Alive | 4845 | 3 |
| TCGA-MJ-A68H | FEMALE | 57.8 | BLACK OR AFRICAN AMERICAN | Alive | 456 | 3 |
| TCGA-DX-A3LS | FEMALE | 78.5 | WHITE | Dead | 275 | 1 |
| TCGA-DX-A1L2 | MALE | 78.6 | WHITE | Alive | 2586 | 1 |
| TCGA-FX-A48G | FEMALE | 56.9 | WHITE | Dead | 605 | 3 |
| TCGA-3B-A9HJ | MALE | 68.4 | WHITE | Alive | 1104 | 1 |
| TCGA-DX-AB35 | MALE | 65.8 | WHITE | Alive | 928 | 2 |
| TCGA-K1-A3PO | MALE | 42.4 | WHITE | Alive | 1622 | 3 |
| TCGA-DX-A6YZ | MALE | 60 | WHITE | Alive | 2625 | 1 |
| TCGA-SG-A6Z4 | MALE | 47.6 | WHITE | Alive | 576 | 1 |
| TCGA-DX-A240 | MALE | 51.3 | WHITE | Alive | 1834 | 1 |
| TCGA-DX-A6YR | MALE | 75.2 | WHITE | Alive | 1247 | 1 |
| TCGA-DX-A7ET | MALE | 71.8 | BLACK OR AFRICAN AMERICAN | Alive | 463 | 1 |
| TCGA-DX-A23Y | MALE | 80.2 | WHITE | Dead | 191 | 1 |
| TCGA-DX-A1L3 | FEMALE | 60.9 | WHITE | Dead | 1020 | 1 |
| TCGA-SI-A71Q | FEMALE | 34.4 | BLACK OR AFRICAN AMERICAN | Alive | 996 | 2 |
| TCGA-DX-AB2L | MALE | 35.5 | WHITE | Alive | 2007 | 1 |
| TCGA-DX-A1KU | MALE | 82.3 | WHITE | Dead | 427 | 1 |
| TCGA-PC-A5DL | MALE | 67.8 | WHITE | Dead | 1142 | 3 |
| TCGA-3B-A9HP | FEMALE | 57.8 | WHITE | Dead | 1627 | 3 |
| TCGA-DX-AB3A | MALE | 80.1 | WHITE | Dead | 978 | 2 |
| TCGA-DX-A6BA | FEMALE | 53.3 | WHITE | Alive | 2515 | 3 |
| TCGA-DX-A2J4 | MALE | 60.6 | WHITE | Alive | 2408 | 1 |
| TCGA-DX-A6YQ | FEMALE | 86.1 | Unknown | Dead | 93 | 1 |
| TCGA-IE-A6BZ | FEMALE | 65.1 | WHITE | Alive | 605 | 1 |
| TCGA-DX-A1KW | MALE | 67.5 | WHITE | Dead | 995 | 2 |
| TCGA-SI-A71P | MALE | 50.3 | WHITE | Alive | 771 | 1 |
| TCGA-QC-A7B5 | MALE | 75.5 | WHITE | Alive | 398 | 1 |
| TCGA-VT-A80J | FEMALE | 49.1 | WHITE | Alive | 796 | 1 |
| TCGA-PC-A5DM | MALE | 73.1 | WHITE | Dead | 193 | 1 |
| TCGA-N1-A6IA | FEMALE | 41.4 | BLACK OR AFRICAN AMERICAN | Dead | 224 | 1 |
| TCGA-PC-A5DO | FEMALE | 59.2 | WHITE | Dead | 2464 | 3 |
| TCGA-DX-A3LW | MALE | 43.8 | WHITE | Alive | 4150 | 2 |
| TCGA-X6-A7WD | FEMALE | 63.9 | WHITE | Alive | 938 | 3 |
| TCGA-WK-A8XO | FEMALE | 66.3 | WHITE | Dead | 363 | 1 |
| TCGA-VT-A80G | MALE | 66.1 | WHITE | Alive | 326 | 1 |
| TCGA-DX-AB3C | MALE | 27.5 | WHITE | Alive | 2169 | 2 |
| TCGA-DX-A23U | MALE | 81.9 | WHITE | Dead | 2324 | 1 |
| TCGA-DX-A3UB | MALE | 54.4 | WHITE | Alive | 2247 | 3 |
| TCGA-DX-AB2V | MALE | 81.4 | WHITE | Alive | 3156 | 1 |
| TCGA-K1-A42X | FEMALE | 62.1 | WHITE | Alive | 3765 | 3 |
| TCGA-DX-A1KX | FEMALE | 67.9 | WHITE | Alive | 2087 | 1 |
| TCGA-X6-A8C3 | FEMALE | 59.6 | ASIAN | Alive | 583 | 1 |
| TCGA-DX-A1KZ | MALE | 66.7 | WHITE | Alive | 1372 | 1 |
| TCGA-KD-A5QS | FEMALE | 55.9 | WHITE | Dead | 1941 | 1 |
| TCGA-DX-A48R | MALE | 41.4 | WHITE | Alive | 805 | 3 |
| TCGA-DX-A1L1 | MALE | 60.9 | WHITE | Dead | 1649 | 1 |
| TCGA-X6-A7WC | MALE | 74.7 | WHITE | Alive | 1379 | 1 |
| TCGA-3B-A9HL | MALE | 67.5 | WHITE | Dead | 599 | 1 |
| TCGA-DX-A3U8 | MALE | 43.2 | Unknown | Dead | 138 | 1 |
| TCGA-FX-A3RE | FEMALE | 65.1 | WHITE | Alive | 660 | 2 |
| TCGA-IE-A4EJ | FEMALE | 84.5 | WHITE | Alive | 98 | 1 |
| TCGA-FX-A8OO | FEMALE | 31.2 | WHITE | Dead | 245 | 2 |
| TCGA-DX-A48N | MALE | 47.6 | WHITE | Alive | 1073 | 2 |
| TCGA-X6-A7W8 | MALE | 90 | WHITE | Dead | 24 | 1 |
| TCGA-WK-A8XT | FEMALE | 41 | WHITE | Dead | 1262 | 2 |
| TCGA-DX-AB2T | FEMALE | 54.1 | BLACK OR AFRICAN AMERICAN | Alive | 2369 | 1 |
| TCGA-DX-AB2E | MALE | 60.6 | WHITE | Alive | 541 | 1 |
| TCGA-QQ-A5VD | MALE | 52.1 | WHITE | Alive | 1129 | 3 |
| TCGA-3B-A9HS | MALE | 60.1 | WHITE | Dead | 1366 | 1 |
| TCGA-KF-A41W | FEMALE | 71.1 | WHITE | Alive | 356 | 1 |
| TCGA-DX-A7EI | FEMALE | 78.4 | WHITE | Dead | 1049 | 2 |
| TCGA-X2-A95T | FEMALE | 49.5 | WHITE | Alive | 1927 | 3 |
| TCGA-IF-A4AJ | MALE | 80.7 | WHITE | Dead | 767 | 3 |
| TCGA-DX-A6B8 | MALE | 80.9 | WHITE | Dead | 485 | 1 |
| TCGA-X6-A8C7 | FEMALE | 24.9 | WHITE | Alive | 896 | 2 |
| TCGA-DX-A23R | FEMALE | 56 | WHITE | Alive | 2271 | 2 |
| TCGA-FX-A3NJ | MALE | 54.5 | WHITE | Alive | 947 | 3 |
| TCGA-LI-A67I | FEMALE | 75.8 | WHITE | Alive | 922 | 1 |
| TCGA-WK-A8XX | MALE | 70.4 | WHITE | Dead | 1164 | 3 |
| TCGA-DX-A8BK | FEMALE | 61.4 | WHITE | Alive | 1885 | 1 |
| TCGA-DX-A7EM | MALE | 61.7 | WHITE | Alive | 1892 | 3 |
| TCGA-DX-A1KY | MALE | 66.5 | WHITE | Dead | 391 | 2 |
| TCGA-DX-A3UD | MALE | 56.7 | WHITE | Alive | 1534 | 3 |
| TCGA-K1-A3PN | FEMALE | 64.7 | WHITE | Alive | 3080 | 3 |
| TCGA-DX-A3M2 | MALE | 59.4 | WHITE | Alive | 1055 | 1 |
| TCGA-DX-AB2O | FEMALE | 78.8 | WHITE | Alive | 2619 | 1 |
| TCGA-WK-A8XY | FEMALE | 53.7 | WHITE | Dead | 1991 | 3 |
| TCGA-DX-A2J1 | MALE | 54 | WHITE | Alive | 2901 | 1 |
| TCGA-QC-A6FX | MALE | 68.8 | WHITE | Alive | 637 | 1 |
| TCGA-DX-AB2G | MALE | 36.3 | WHITE | Alive | 478 | 1 |
| TCGA-DX-A8BP | MALE | 86 | WHITE | Alive | 428 | 1 |
| TCGA-DX-A1L0 | MALE | 58.7 | WHITE | Dead | 564 | 2 |
| TCGA-UE-A6QT | FEMALE | 50.7 | ASIAN | Alive | 402 | 1 |
| TCGA-MB-A5Y8 | FEMALE | 60.6 | WHITE | Alive | 773 | 1 |
| TCGA-FX-A76Y | FEMALE | 49.8 | WHITE | Alive | 471 | 3 |
| TCGA-X6-A8C5 | FEMALE | 64 | BLACK OR AFRICAN AMERICAN | Alive | 1547 | 3 |
| TCGA-QQ-A5VB | FEMALE | 53.8 | WHITE | Dead | 1478 | 1 |
| TCGA-IW-A3M4 | FEMALE | 48.6 | WHITE | Alive | 2615 | 3 |
| TCGA-DX-A8BV | MALE | 57.5 | WHITE | Dead | 1845 | 1 |
| TCGA-DX-AB3B | FEMALE | 28.3 | WHITE | Alive | 1548 | 2 |
| TCGA-DX-A23V | FEMALE | 58 | WHITE | Dead | 2034 | 1 |
| TCGA-DX-AB2J | FEMALE | 71.7 | WHITE | Dead | 1235 | 2 |
| TCGA-DX-A6BG | MALE | 51.2 | WHITE | Alive | 1121 | 1 |
| TCGA-DX-A48P | FEMALE | 54.2 | WHITE | Alive | 1400 | 3 |
| TCGA-DX-A7ES | FEMALE | 50.8 | WHITE | Alive | 610 | 1 |
| TCGA-DX-A48J | MALE | 44.6 | BLACK OR AFRICAN AMERICAN | Dead | 296 | 3 |
| TCGA-MB-A5Y9 | MALE | 90.1 | WHITE | Alive | 0 | 1 |
| TCGA-DX-A48O | FEMALE | 64 | WHITE | Alive | 1605 | 3 |
| TCGA-QQ-A8VB | FEMALE | 68.3 | WHITE | Alive | 5723 | 2 |
| TCGA-DX-A8BU | MALE | 58.8 | WHITE | Dead | 1116 | 1 |
| TCGA-IE-A4EH | FEMALE | 35.3 | WHITE | Alive | 819 | 3 |
| TCGA-IS-A3KA | FEMALE | 73.4 | WHITE | Dead | 413 | 3 |
| TCGA-WP-A9GB | FEMALE | NA | WHITE | Dead | 261 | 3 |
| TCGA-DX-A8BM | MALE | 60.7 | WHITE | Alive | 1456 | 1 |
| TCGA-DX-A6Z2 | FEMALE | 82.3 | WHITE | Alive | 2830 | 3 |
| TCGA-DX-A2IZ | MALE | 60.9 | WHITE | Alive | 261 | 2 |
| TCGA-SI-AA8C | FEMALE | 20.6 | WHITE | Alive | 597 | 2 |
| TCGA-DX-AB2Z | FEMALE | 87.1 | WHITE | Alive | 1092 | 1 |
| TCGA-QQ-A5V2 | MALE | 42.2 | WHITE | Alive | 56 | 1 |
| TCGA-SI-AA8B | FEMALE | 80.3 | WHITE | Dead | 262 | 1 |
| TCGA-IE-A3OV | MALE | 42.8 | WHITE | Dead | 2448 | 3 |
| TCGA-DX-A8BT | FEMALE | 63.6 | WHITE | Alive | 1230 | 1 |
| TCGA-PC-A5DN | FEMALE | 77.9 | WHITE | Dead | 1175 | 2 |
| TCGA-MB-A8JK | MALE | 49.8 | WHITE | Alive | 661 | 1 |
| TCGA-VT-AB3D | MALE | 71.5 | WHITE | Alive | 379 | 1 |
| TCGA-KD-A5QT | FEMALE | 69.2 | Not Evaluated | Alive | 499 | 3 |
| TCGA-DX-A8BG | FEMALE | 83.1 | WHITE | Alive | 546 | 1 |
| TCGA-DX-A8BR | FEMALE | 63.8 | WHITE | Alive | 4573 | 1 |
| TCGA-HB-A3YV | MALE | 75.6 | WHITE | Dead | 139 | 2 |
| TCGA-DX-A3UE | FEMALE | 66.8 | WHITE | Dead | 1061 | 3 |
| TCGA-K1-A6RV | MALE | 67.7 | WHITE | Alive | 158 | 1 |
| TCGA-WK-A8XZ | FEMALE | 56.1 | WHITE | Dead | 1722 | 3 |
| TCGA-3B-A9HX | FEMALE | 70.2 | WHITE | Alive | 1303 | 3 |
| TCGA-SG-A849 | MALE | 78.1 | WHITE | Alive | 533 | 1 |
| TCGA-SI-A71O | MALE | 29.3 | WHITE | Dead | 694 | 1 |
| TCGA-DX-A1L4 | MALE | 60.8 | WHITE | Alive | 2036 | 1 |
| TCGA-IS-A3K7 | FEMALE | 63.1 | WHITE | Alive | 5204 | 1 |
| TCGA-DX-AB36 | MALE | 86.9 | WHITE | Dead | 1168 | 1 |
| TCGA-DX-A7EO | FEMALE | 20.8 | WHITE | Alive | 4627 | 2 |
| TCGA-DX-A6BH | MALE | 34.9 | WHITE | Alive | 821 | 1 |
| TCGA-Z4-A8JB | FEMALE | 25 | WHITE | Alive | 133 | 1 |
| TCGA-3B-A9HZ | MALE | 66.9 | WHITE | Alive | 1170 | 3 |
| TCGA-DX-A6B9 | FEMALE | 45.4 | BLACK OR AFRICAN AMERICAN | Alive | 1138 | 3 |
| TCGA-IF-A4AK | FEMALE | 82.6 | WHITE | Alive | 1590 | 3 |
| TCGA-DX-AB37 | FEMALE | 73.1 | WHITE | Dead | 2575 | 1 |
| TCGA-DX-A6BF | FEMALE | 82.4 | WHITE | Alive | 972 | 1 |
| TCGA-DX-A7EF | FEMALE | 88.5 | WHITE | Dead | 570 | 1 |
| TCGA-X9-A973 | MALE | 55.2 | WHITE | Alive | 1108 | 2 |
| TCGA-DX-A3LU | MALE | 60 | WHITE | Alive | 2641 | 1 |
| TCGA-JV-A5VE | FEMALE | 60.1 | BLACK OR AFRICAN AMERICAN | Alive | 499 | 3 |
| TCGA-PT-A8TR | FEMALE | 57.5 | WHITE | Alive | 813 | 2 |
| TCGA-Z4-AAPF | FEMALE | 35.5 | WHITE | Alive | 485 | 2 |
| TCGA-3B-A9I1 | FEMALE | 56.5 | BLACK OR AFRICAN AMERICAN | Dead | 567 | 3 |
| TCGA-QQ-A8VH | FEMALE | 31.8 | WHITE | Dead | 320 | 2 |
| TCGA-DX-A23T | FEMALE | 40.8 | WHITE | Dead | 160 | 1 |
| TCGA-X6-A8C2 | MALE | 56.2 | WHITE | Alive | 1070 | 1 |
| TCGA-3B-A9HV | FEMALE | 48.3 | WHITE | Dead | 437 | 3 |
| TCGA-DX-A3UA | FEMALE | 47.5 | WHITE | Dead | 325 | 2 |
| TCGA-DX-AB2W | FEMALE | 62.9 | WHITE | Alive | 3238 | 1 |
| TCGA-KD-A5QU | FEMALE | 41.1 | WHITE | Dead | 1073 | 3 |
| TCGA-QQ-A5VC | FEMALE | 64 | WHITE | Alive | 1092 | 1 |
| TCGA-DX-A8BJ | MALE | 77 | WHITE | Dead | 284 | 1 |
| TCGA-HB-A2OT | FEMALE | 78.4 | BLACK OR AFRICAN AMERICAN | Alive | 2057 | 1 |
| TCGA-DX-A6B7 | MALE | 55.8 | WHITE | Alive | 1143 | 3 |
| TCGA-FX-A3NK | MALE | 53.5 | WHITE | Alive | 505 | 2 |
| TCGA-DX-A7EL | FEMALE | 56.9 | WHITE | Alive | 2291 | 3 |
| TCGA-3B-A9I0 | MALE | 33.3 | WHITE | Alive | 1096 | 3 |
| TCGA-PC-A5DK | MALE | 63.1 | WHITE | Alive | 3740 | 3 |
| TCGA-QC-AA9N | FEMALE | 53.1 | BLACK OR AFRICAN AMERICAN | Alive | 537 | 2 |
| TCGA-DX-A3U5 | MALE | 52.3 | WHITE | Alive | 969 | 1 |
| TCGA-DX-AB2Q | FEMALE | 65.1 | WHITE | Alive | 2030 | 1 |
| TCGA-DX-A7EN | FEMALE | 53.7 | WHITE | Alive | 923 | 3 |
| TCGA-QQ-A8VG | MALE | 52.9 | WHITE | Dead | 1970 | 1 |
| TCGA-X9-A971 | FEMALE | 52.6 | WHITE | Alive | 831 | 3 |
| TCGA-DX-A7EU | FEMALE | 80 | WHITE | Alive | 721 | 1 |
| TCGA-Z4-AAPG | FEMALE | 64.1 | WHITE | Alive | 486 | 1 |
| TCGA-DX-A48L | FEMALE | 49.6 | WHITE | Dead | 738 | 3 |
| TCGA-3B-A9HI | MALE | 68.4 | WHITE | Alive | 1521 | 1 |
| TCGA-DX-A8BZ | FEMALE | 79 | BLACK OR AFRICAN AMERICAN | Dead | 200 | 1 |
| TCGA-3B-A9HY | MALE | 49.6 | WHITE | Alive | 314 | 3 |
| TCGA-3B-A9HU | FEMALE | 53.9 | BLACK OR AFRICAN AMERICAN | Alive | 768 | 3 |
| TCGA-DX-AB2S | FEMALE | 54 | WHITE | Alive | 280 | 1 |
| TCGA-DX-A3U6 | FEMALE | 63.5 | WHITE | Dead | 1088 | 1 |
| TCGA-DX-A7EQ | MALE | 72.6 | WHITE | Dead | 897 | 2 |
| TCGA-DX-A2J0 | MALE | 61.3 | WHITE | Dead | 295 | 2 |
| TCGA-Z4-A9VC | MALE | 37.2 | WHITE | Alive | 407 | 2 |
| TCGA-SG-A6Z7 | FEMALE | 44.4 | WHITE | Dead | 118 | 2 |
| TCGA-FX-A3TO | MALE | 87.3 | WHITE | Alive | 618 | 1 |
| TCGA-K1-A42W | FEMALE | 65.5 | WHITE | Alive | 1891 | 3 |
| TCGA-JV-A5VF | FEMALE | 69.9 | BLACK OR AFRICAN AMERICAN | Dead | 32 | 1 |
| TCGA-DX-A3UF | FEMALE | 65.7 | WHITE | Dead | 2599 | 3 |
| TCGA-DX-A3UC | FEMALE | 37.7 | WHITE | Alive | 2085 | 3 |
| TCGA-DX-A7ER | MALE | 69.5 | WHITE | Dead | 1466 | 2 |
| TCGA-JV-A75J | FEMALE | 43.8 | WHITE | Dead | 119 | 2 |
| TCGA-DX-A48K | MALE | 65.1 | WHITE | Alive | 1399 | 1 |
| TCGA-DX-A8BX | MALE | 53 | WHITE | Dead | 591 | 1 |
| TCGA-QQ-A8VD | MALE | 56.8 | WHITE | Alive | 3964 | 1 |
| TCGA-IW-A3M5 | FEMALE | 76.4 | WHITE | Dead | 180 | 2 |
| TCGA-QQ-A5VA | FEMALE | 60.9 | WHITE | Dead | 550 | 1 |
| TCGA-DX-A48U | MALE | 57.1 | WHITE | Alive | 3310 | 3 |
| TCGA-IE-A4EI | FEMALE | 67.6 | WHITE | Alive | 594 | 3 |
| TCGA-DX-AB2P | MALE | 79.8 | WHITE | Dead | 367 | 1 |
| TCGA-WK-A8XS | FEMALE | 59 | WHITE | Alive | 2579 | 3 |
| TCGA-MO-A47R | FEMALE | 76 | ASIAN | Dead | 318 | 2 |
| TCGA-DX-AB32 | MALE | 51 | WHITE | Alive | 3102 | 1 |
| TCGA-HB-A5W3 | FEMALE | 58.7 | WHITE | Dead | 384 | 3 |
| TCGA-MJ-A68J | FEMALE | 55.1 | BLACK OR AFRICAN AMERICAN | Alive | 547 | 1 |
| TCGA-DX-A6Z0 | FEMALE | 78.9 | WHITE | Dead | 1953 | 1 |
| TCGA-DX-AB2H | FEMALE | 81 | WHITE | Alive | 482 | 2 |
| TCGA-3B-A9HQ | FEMALE | 66.8 | WHITE | Alive | 2085 | 3 |
| TCGA-IE-A4EK | MALE | 54.3 | WHITE | Alive | 42 | 3 |
| TCGA-DX-AB2F | FEMALE | 76.3 | WHITE | Alive | 365 | 2 |
| TCGA-X6-A8C6 | MALE | 55 | WHITE | Dead | 1067 | 1 |
| TCGA-IS-A3K6 | FEMALE | 55.4 | WHITE | Dead | 858 | 3 |
| TCGA-HS-A5N7 | FEMALE | 67.9 | Not Evaluated | Alive | 1013 | 3 |
| TCGA-QQ-A5V9 | MALE | 76.6 | WHITE | Dead | 1424 | 1 |
| TCGA-DX-AATS | MALE | 72.9 | WHITE | Dead | 35 | 2 |
| TCGA-MO-A47P | FEMALE | 37.7 | ASIAN | Alive | 352 | 3 |
| TCGA-DX-A8BO | FEMALE | 67.2 | WHITE | Alive | 1240 | 1 |
| TCGA-PC-A5DP | FEMALE | 72.4 | WHITE | Dead | 550 | 3 |
| TCGA-DX-A3M1 | MALE | 79.3 | WHITE | Alive | 1359 | 2 |
| TCGA-WK-A8XQ | MALE | 57.2 | WHITE | Dead | 146 | 2 |
