# Supplementary material for: Integrative Clustering Reveals a Novel Subtype of Soft Tissue Sarcoma With Poor Prognosis
Source: Front Genet. 2020 Feb 17;11:69. doi: 10.3389/fgene.2020.00069 (PMC7038822; doi:10.3389/fgene.2020.00069)
Supplement: Supplementary file 3 [file Table_2.docx]

Supplementary Table 2: Mutation rate of genes in each sub-cluster

|  | percent_C1 | percent_C2 | percent_C3 | P_value |
| --- | --- | --- | --- | --- |
| TTN | 0.080972 | 0.040486 | 0.032389 | 0.34578 |
| RNF212 | 0.040486 | 0.032389 | 0.016194 | 0.068687 |
| TP53 | 0.17004 | 0.020243 | 0.149798 | 0.000239 |
| WDFY3 | 0.016194 | 0.020243 | 0.004049 | 0.036985 |
| MT-CO2 | 0.020243 | 0.020243 | 0.004049 | 0.065537 |
| MT-RNR2 | 0.036437 | 0.020243 | 0 | 0.21593 |
| HELZ2 | 0.040486 | 0.020243 | 0.008097 | 0.4473 |
| MUC16 | 0.064777 | 0.020243 | 0.020243 | 1 |
| FAT2 | 0.004049 | 0.016194 | 0.004049 | 0.014773 |
| XYLB | 0.012146 | 0.016194 | 0.004049 | 0.077219 |
| ATRX | 0.101215 | 0.016194 | 0.044534 | 0.153067 |
| ANK3 | 0.020243 | 0.016194 | 0.004049 | 0.204064 |
| KRTAP5-5 | 0.020243 | 0.016194 | 0.004049 | 0.204064 |
| SPEG | 0.02834 | 0.016194 | 0.004049 | 0.382325 |
| SYNE1 | 0.036437 | 0.016194 | 0.004049 | 0.587826 |
| GPR98 | 0.032389 | 0.016194 | 0.008097 | 0.587826 |
| NEB | 0.036437 | 0.016194 | 0.016194 | 0.900741 |
| ADCY2 | 0.008097 | 0.012146 | 0 | 0.080964 |
| ANGEL2 | 0.008097 | 0.012146 | 0 | 0.080964 |
| APC | 0.008097 | 0.012146 | 0 | 0.080964 |
| LOC100133091 | 0.004049 | 0.012146 | 0.004049 | 0.080964 |
| LOC100508046 | 0.004049 | 0.012146 | 0.004049 | 0.080964 |
| OS9 | 0.004049 | 0.012146 | 0.004049 | 0.080964 |
| ATXN7L1 | 0.012146 | 0.012146 | 0 | 0.163502 |
| BAZ2A | 0.012146 | 0.012146 | 0 | 0.163502 |
| PIK3CA | 0.012146 | 0.012146 | 0 | 0.163502 |
| SCN8A | 0.012146 | 0.012146 | 0 | 0.163502 |
| SORCS3 | 0.012146 | 0.012146 | 0 | 0.163502 |
| TRDN | 0.012146 | 0.012146 | 0 | 0.163502 |
| ACADVL | 0.016194 | 0.012146 | 0 | 0.269421 |
| DCAF8L2 | 0.016194 | 0.012146 | 0 | 0.269421 |
| SLIT3 | 0.016194 | 0.012146 | 0 | 0.269421 |
| ENSG00000221280 | 0.012146 | 0.012146 | 0.004049 | 0.269421 |
| TCHH | 0.008097 | 0.012146 | 0.008097 | 0.269421 |
| FAM230C | 0.004049 | 0.012146 | 0.012146 | 0.269421 |
| NTM | 0.020243 | 0.012146 | 0 | 0.390503 |
| CACNA1A | 0.016194 | 0.012146 | 0.004049 | 0.390503 |
| HSPG2 | 0.016194 | 0.012146 | 0.004049 | 0.390503 |
| PRKDC | 0.012146 | 0.012146 | 0.008097 | 0.390503 |
| CABP1 | 0.024291 | 0.012146 | 0 | 0.519239 |
| WWC1 | 0.012146 | 0.012146 | 0.012146 | 0.519239 |
| RNF213 | 0.02834 | 0.012146 | 0 | 0.649698 |
| CSMD3 | 0.024291 | 0.012146 | 0.004049 | 0.649698 |
| FLG | 0.020243 | 0.012146 | 0.008097 | 0.649698 |
| LOR | 0.020243 | 0.012146 | 0.008097 | 0.649698 |
| UNC13C | 0.020243 | 0.012146 | 0.008097 | 0.649698 |
| LOC101927345 | 0.02834 | 0.012146 | 0.004049 | 0.777583 |
| FCGBP | 0.020243 | 0.012146 | 0.012146 | 0.777583 |
| MYADML | 0.020243 | 0.012146 | 0.012146 | 0.777583 |
| ADAM21P1 | 0.016194 | 0.012146 | 0.016194 | 0.777583 |
| DNM1P47 | 0.052632 | 0.012146 | 0.016194 | 0.819711 |
| PCLO | 0.052632 | 0.012146 | 0.016194 | 0.819711 |
| OBSCN | 0.048583 | 0.012146 | 0.016194 | 0.907612 |
| RYR1 | 0.032389 | 0.012146 | 0.016194 | 1 |
| XIST | 0.02834 | 0.012146 | 0.012146 | 1 |
| MUC12 | 0.020243 | 0.012146 | 0.02834 | 1 |
| MUC5B | 0.052632 | 0.012146 | 0.008097 | 1 |
| FAT1 | 0.040486 | 0.012146 | 0.004049 | 1 |
| FAT3 | 0.036437 | 0.012146 | 0.008097 | 1 |
| NCOR2 | 0.024291 | 0.012146 | 0.020243 | 1 |
| MALAT1 | 0.020243 | 0.012146 | 0.024291 | 1 |
| DNAH8 | 0.048583 | 0.008097 | 0.016194 | 0.536973 |
| NRXN1 | 0.02834 | 0.008097 | 0.036437 | 0.536973 |
| AKAP9 | 0.012146 | 0.008097 | 0 | 0.546322 |
| AMY2B | 0.012146 | 0.008097 | 0 | 0.546322 |
| ARHGAP30 | 0.012146 | 0.008097 | 0 | 0.546322 |
| BRCA2 | 0.012146 | 0.008097 | 0 | 0.546322 |
| CXADRP3 | 0.012146 | 0.008097 | 0 | 0.546322 |
| FGFR4 | 0.012146 | 0.008097 | 0 | 0.546322 |
| FMNL2 | 0.012146 | 0.008097 | 0 | 0.546322 |
| KRTAP9-9 | 0.012146 | 0.008097 | 0 | 0.546322 |
| MGA | 0.012146 | 0.008097 | 0 | 0.546322 |
| MYH13 | 0.012146 | 0.008097 | 0 | 0.546322 |
| NBPF10 | 0.012146 | 0.008097 | 0 | 0.546322 |
| NWD1 | 0.012146 | 0.008097 | 0 | 0.546322 |
| PRKXP1 | 0.012146 | 0.008097 | 0 | 0.546322 |
| RDH13 | 0.012146 | 0.008097 | 0 | 0.546322 |
| SIGLEC7 | 0.012146 | 0.008097 | 0 | 0.546322 |
| SP140 | 0.012146 | 0.008097 | 0 | 0.546322 |
| TET2 | 0.012146 | 0.008097 | 0 | 0.546322 |
| WRN | 0.012146 | 0.008097 | 0 | 0.546322 |
| ZNF608 | 0.012146 | 0.008097 | 0 | 0.546322 |
| ABCC2 | 0.008097 | 0.008097 | 0.004049 | 0.546322 |
| AFAP1L2 | 0.008097 | 0.008097 | 0.004049 | 0.546322 |
| ANO4 | 0.008097 | 0.008097 | 0.004049 | 0.546322 |
| COL7A1 | 0.008097 | 0.008097 | 0.004049 | 0.546322 |
| DIP2B | 0.008097 | 0.008097 | 0.004049 | 0.546322 |
| ELN | 0.008097 | 0.008097 | 0.004049 | 0.546322 |
| FAM120C | 0.008097 | 0.008097 | 0.004049 | 0.546322 |
| KMT2E | 0.008097 | 0.008097 | 0.004049 | 0.546322 |
| MET | 0.008097 | 0.008097 | 0.004049 | 0.546322 |
| MUC6 | 0.008097 | 0.008097 | 0.004049 | 0.546322 |
| SGIP1 | 0.008097 | 0.008097 | 0.004049 | 0.546322 |
| SPATA42 | 0.008097 | 0.008097 | 0.004049 | 0.546322 |
| TPRXL | 0.008097 | 0.008097 | 0.004049 | 0.546322 |
| TRPC7 | 0.008097 | 0.008097 | 0.004049 | 0.546322 |
| TTC6 | 0.008097 | 0.008097 | 0.004049 | 0.546322 |
| ZMAT1 | 0.008097 | 0.008097 | 0.004049 | 0.546322 |
| CD3EAP | 0.004049 | 0.008097 | 0.008097 | 0.546322 |
| CEP290 | 0.004049 | 0.008097 | 0.008097 | 0.546322 |
| HERC2P4 | 0.004049 | 0.008097 | 0.008097 | 0.546322 |
| HLA-V | 0.004049 | 0.008097 | 0.008097 | 0.546322 |
| MEG3 | 0.004049 | 0.008097 | 0.008097 | 0.546322 |
| NPAP1 | 0.004049 | 0.008097 | 0.008097 | 0.546322 |
| SPTBN2 | 0.004049 | 0.008097 | 0.008097 | 0.546322 |
| TTLL11 | 0.004049 | 0.008097 | 0.008097 | 0.546322 |
| VPS13C | 0.004049 | 0.008097 | 0.008097 | 0.546322 |
| ENSG00000221060 | 0 | 0.008097 | 0.012146 | 0.546322 |
| MACF1 | 0.040486 | 0.008097 | 0.020243 | 0.60972 |
| CHD2 | 0.016194 | 0.008097 | 0 | 0.727182 |
| DNM1P51 | 0.016194 | 0.008097 | 0 | 0.727182 |
| FAM135B | 0.016194 | 0.008097 | 0 | 0.727182 |
| GALNT6 | 0.016194 | 0.008097 | 0 | 0.727182 |
| LHCGR | 0.016194 | 0.008097 | 0 | 0.727182 |
| PALM2-AKAP2 | 0.016194 | 0.008097 | 0 | 0.727182 |
| PCDHB6 | 0.016194 | 0.008097 | 0 | 0.727182 |
| PRRC2A | 0.016194 | 0.008097 | 0 | 0.727182 |
| SMG6 | 0.016194 | 0.008097 | 0 | 0.727182 |
| SYNE2 | 0.016194 | 0.008097 | 0 | 0.727182 |
| SYNM | 0.016194 | 0.008097 | 0 | 0.727182 |
| WASH3P | 0.016194 | 0.008097 | 0 | 0.727182 |
| CEP350 | 0.012146 | 0.008097 | 0.004049 | 0.727182 |
| DCHS1 | 0.012146 | 0.008097 | 0.004049 | 0.727182 |
| DNAH10 | 0.012146 | 0.008097 | 0.004049 | 0.727182 |
| DPP6 | 0.012146 | 0.008097 | 0.004049 | 0.727182 |
| ENSG00000226145 | 0.012146 | 0.008097 | 0.004049 | 0.727182 |
| HERC2 | 0.012146 | 0.008097 | 0.004049 | 0.727182 |
| KIF26A | 0.012146 | 0.008097 | 0.004049 | 0.727182 |
| KMT2C | 0.012146 | 0.008097 | 0.004049 | 0.727182 |
| LRP1 | 0.012146 | 0.008097 | 0.004049 | 0.727182 |
| MYH14 | 0.012146 | 0.008097 | 0.004049 | 0.727182 |
| OR4C5 | 0.012146 | 0.008097 | 0.004049 | 0.727182 |
| PDE3B | 0.012146 | 0.008097 | 0.004049 | 0.727182 |
| PDZD2 | 0.012146 | 0.008097 | 0.004049 | 0.727182 |
| PRX | 0.012146 | 0.008097 | 0.004049 | 0.727182 |
| SPATA31D1 | 0.012146 | 0.008097 | 0.004049 | 0.727182 |
| SRGAP3 | 0.012146 | 0.008097 | 0.004049 | 0.727182 |
| AQP4 | 0.008097 | 0.008097 | 0.008097 | 0.727182 |
| BOD1L1 | 0.008097 | 0.008097 | 0.008097 | 0.727182 |
| CDH23 | 0.008097 | 0.008097 | 0.008097 | 0.727182 |
| ENSG00000229334 | 0.008097 | 0.008097 | 0.008097 | 0.727182 |
| F8 | 0.008097 | 0.008097 | 0.008097 | 0.727182 |
| FLNA | 0.008097 | 0.008097 | 0.008097 | 0.727182 |
| KRT1 | 0.008097 | 0.008097 | 0.008097 | 0.727182 |
| LRP2 | 0.036437 | 0.008097 | 0.016194 | 0.779933 |
| ENSG00000225411 | 0.02834 | 0.008097 | 0.020243 | 0.878046 |
| ABCB5 | 0.020243 | 0.008097 | 0 | 0.892333 |
| DSG2 | 0.020243 | 0.008097 | 0 | 0.892333 |
| PIK3C2G | 0.020243 | 0.008097 | 0 | 0.892333 |
| PRRC2B | 0.020243 | 0.008097 | 0 | 0.892333 |
| CASZ1 | 0.016194 | 0.008097 | 0.004049 | 0.892333 |
| COL1A1 | 0.016194 | 0.008097 | 0.004049 | 0.892333 |
| FAM47C | 0.016194 | 0.008097 | 0.004049 | 0.892333 |
| FBN2 | 0.016194 | 0.008097 | 0.004049 | 0.892333 |
| IRF5 | 0.016194 | 0.008097 | 0.004049 | 0.892333 |
| LRRK2 | 0.016194 | 0.008097 | 0.004049 | 0.892333 |
| ZNF521 | 0.016194 | 0.008097 | 0.004049 | 0.892333 |
| ARHGEF11 | 0.012146 | 0.008097 | 0.008097 | 0.892333 |
| DNAH3 | 0.012146 | 0.008097 | 0.008097 | 0.892333 |
| FAM157A | 0.012146 | 0.008097 | 0.008097 | 0.892333 |
| KIT | 0.012146 | 0.008097 | 0.008097 | 0.892333 |
| MAML3 | 0.012146 | 0.008097 | 0.008097 | 0.892333 |
| NINL | 0.012146 | 0.008097 | 0.008097 | 0.892333 |
| PDE4DIP | 0.012146 | 0.008097 | 0.008097 | 0.892333 |
| SETX | 0.012146 | 0.008097 | 0.008097 | 0.892333 |
| ENSG00000272231 | 0.008097 | 0.008097 | 0.012146 | 0.892333 |
| FCRL5 | 0.008097 | 0.008097 | 0.012146 | 0.892333 |
| KANK3 | 0.008097 | 0.008097 | 0.012146 | 0.892333 |
| LOC101928729 | 0.008097 | 0.008097 | 0.012146 | 0.892333 |
| RABGGTB | 0.008097 | 0.008097 | 0.012146 | 0.892333 |
| ENSG00000225946 | 0.004049 | 0.008097 | 0.016194 | 0.892333 |
| KRTAP10-9 | 0.004049 | 0.008097 | 0.016194 | 0.892333 |
| PKHD1L1 | 0.032389 | 0.008097 | 0.012146 | 0.98488 |
| DNAH5 | 0.02834 | 0.008097 | 0.016194 | 0.98488 |
| HYDIN | 0.02834 | 0.008097 | 0.016194 | 0.98488 |
| PEG3 | 0.036437 | 0.008097 | 0 | 1 |
| PRIM2 | 0.02834 | 0.008097 | 0.008097 | 1 |
| SCN2A | 0.02834 | 0.008097 | 0.008097 | 1 |
| MYO15A | 0.02834 | 0.008097 | 0 | 1 |
| XIRP2 | 0.02834 | 0.008097 | 0 | 1 |
| CACNA1B | 0.024291 | 0.008097 | 0.004049 | 1 |
| DNAH6 | 0.024291 | 0.008097 | 0.004049 | 1 |
| HMCN1 | 0.024291 | 0.008097 | 0.004049 | 1 |
| TENM2 | 0.024291 | 0.008097 | 0.004049 | 1 |
| VWF | 0.024291 | 0.008097 | 0.004049 | 1 |
| C1orf173 | 0.020243 | 0.008097 | 0.008097 | 1 |
| CCDC30 | 0.020243 | 0.008097 | 0.008097 | 1 |
| CROCCP2 | 0.020243 | 0.008097 | 0.008097 | 1 |
| DOCK2 | 0.020243 | 0.008097 | 0.008097 | 1 |
| ENSG00000203849 | 0.020243 | 0.008097 | 0.008097 | 1 |
| ENSG00000232274 | 0.020243 | 0.008097 | 0.008097 | 1 |
| SCN9A | 0.020243 | 0.008097 | 0.008097 | 1 |
| LRBA | 0.016194 | 0.008097 | 0.012146 | 1 |
| MYO5B | 0.016194 | 0.008097 | 0.012146 | 1 |
| COL11A2 | 0.012146 | 0.008097 | 0.016194 | 1 |
| DNAH17 | 0.012146 | 0.008097 | 0.016194 | 1 |
| DCC | 0.040486 | 0.008097 | 0 | 1 |
| APOB | 0.032389 | 0.008097 | 0.008097 | 1 |
| NF1 | 0.032389 | 0.008097 | 0 | 1 |
| PTPRVP | 0.032389 | 0.008097 | 0 | 1 |
| TMEM132C | 0.02834 | 0.008097 | 0.004049 | 1 |
| CTNND2 | 0.024291 | 0.008097 | 0 | 1 |
| DSCAM | 0.024291 | 0.008097 | 0.008097 | 1 |
| MSH3 | 0.024291 | 0.008097 | 0.016194 | 1 |
| NUMBL | 0.024291 | 0.008097 | 0.008097 | 1 |
| DCHS2 | 0.020243 | 0.008097 | 0.020243 | 1 |
| DNAH14 | 0.020243 | 0.008097 | 0.004049 | 1 |
| ENSG00000215941 | 0.020243 | 0.008097 | 0.012146 | 1 |
| IGFN1 | 0.020243 | 0.008097 | 0.004049 | 1 |
| MROH2B | 0.020243 | 0.008097 | 0.004049 | 1 |
| NOS1 | 0.020243 | 0.008097 | 0.004049 | 1 |
| RTL1 | 0.020243 | 0.008097 | 0.004049 | 1 |
| TNXB | 0.020243 | 0.008097 | 0.012146 | 1 |
| ZAN | 0.020243 | 0.008097 | 0.012146 | 1 |
| ZFHX4 | 0.020243 | 0.008097 | 0.004049 | 1 |
| ANKLE1 | 0.016194 | 0.008097 | 0.008097 | 1 |
| C9orf78 | 0.016194 | 0.008097 | 0.008097 | 1 |
| CELSR2 | 0.016194 | 0.008097 | 0.008097 | 1 |
| EP400 | 0.016194 | 0.008097 | 0.008097 | 1 |
| GOLGA6L4 | 0.016194 | 0.008097 | 0.008097 | 1 |
| GPR158 | 0.016194 | 0.008097 | 0.008097 | 1 |
| HRNR | 0.016194 | 0.008097 | 0.016194 | 1 |
| KIAA1211 | 0.016194 | 0.008097 | 0.016194 | 1 |
| KIAA1217 | 0.016194 | 0.008097 | 0.008097 | 1 |
| PHLDB2 | 0.016194 | 0.008097 | 0.008097 | 1 |
| ADAM33 | 0.012146 | 0.008097 | 0.012146 | 1 |
| CAPZA1 | 0.012146 | 0.008097 | 0.012146 | 1 |
| LOC101927832 | 0.012146 | 0.008097 | 0.012146 | 1 |
| PTPRK | 0.012146 | 0.008097 | 0.012146 | 1 |
| LAMA1 | 0.008097 | 0.008097 | 0.016194 | 1 |
| MUC4 | 0.044534 | 0.004049 | 0.02834 | 0.18584 |
| LRP1B | 0.044534 | 0.004049 | 0.024291 | 0.216442 |
| RYR2 | 0.044534 | 0.004049 | 0.012146 | 0.340739 |
| CSMD1 | 0.024291 | 0.004049 | 0.02834 | 0.395948 |
| DMD | 0.036437 | 0.004049 | 0.012146 | 0.45985 |
| DNAH9 | 0.036437 | 0.004049 | 0.012146 | 0.45985 |
| C12orf55 | 0.032389 | 0.004049 | 0.016194 | 0.45985 |
| CSMD2 | 0.032389 | 0.004049 | 0.012146 | 0.533755 |
| DNAH7 | 0.02834 | 0.004049 | 0.016194 | 0.533755 |
| MT-CO1 | 0.020243 | 0.004049 | 0.024291 | 0.533755 |
| SPHKAP | 0.040486 | 0.004049 | 0 | 0.61913 |
| LOXHD1 | 0.032389 | 0.004049 | 0.008097 | 0.61913 |
| PKHD1 | 0.032389 | 0.004049 | 0.008097 | 0.61913 |
| ABCA13 | 0.024291 | 0.004049 | 0.016194 | 0.61913 |
| MKI67 | 0.036437 | 0.004049 | 0 | 0.717581 |
| DNAH11 | 0.032389 | 0.004049 | 0.004049 | 0.717581 |
| GAPVD1 | 0.032389 | 0.004049 | 0.004049 | 0.717581 |
| CELP | 0.02834 | 0.004049 | 0.008097 | 0.717581 |
| PTPRU | 0.02834 | 0.004049 | 0.008097 | 0.717581 |
| RYR3 | 0.02834 | 0.004049 | 0.008097 | 0.717581 |
| ZDHHC11B | 0.02834 | 0.004049 | 0.008097 | 0.717581 |
| CNTNAP3 | 0.024291 | 0.004049 | 0.012146 | 0.717581 |
| ENSG00000234978 | 0.024291 | 0.004049 | 0.012146 | 0.717581 |
| MAGEC1 | 0.024291 | 0.004049 | 0.012146 | 0.717581 |
| RELN | 0.024291 | 0.004049 | 0.012146 | 0.717581 |
| MUC2 | 0.020243 | 0.004049 | 0.016194 | 0.717581 |
| ROBO3 | 0.020243 | 0.004049 | 0.016194 | 0.717581 |
| CDH12 | 0.02834 | 0.004049 | 0.004049 | 0.830796 |
| RIMS2 | 0.02834 | 0.004049 | 0.004049 | 0.830796 |
| UNC80 | 0.02834 | 0.004049 | 0.004049 | 0.830796 |
| COL5A3 | 0.024291 | 0.004049 | 0.008097 | 0.830796 |
| DSPP | 0.024291 | 0.004049 | 0.008097 | 0.830796 |
| DST | 0.024291 | 0.004049 | 0.008097 | 0.830796 |
| DNHD1 | 0.020243 | 0.004049 | 0.012146 | 0.830796 |
| GPRIN1 | 0.020243 | 0.004049 | 0.012146 | 0.830796 |
| LOC100287042 | 0.020243 | 0.004049 | 0.012146 | 0.830796 |
| PLXNA2 | 0.020243 | 0.004049 | 0.012146 | 0.830796 |
| ARAP2 | 0.016194 | 0.004049 | 0.016194 | 0.830796 |
| CASP8AP2 | 0.016194 | 0.004049 | 0.016194 | 0.830796 |
| ENSG00000216020 | 0.016194 | 0.004049 | 0.016194 | 0.830796 |
| SHROOM4 | 0.016194 | 0.004049 | 0.016194 | 0.830796 |
| DNAH2 | 0.012146 | 0.004049 | 0.020243 | 0.830796 |
| FBN3 | 0.02834 | 0.004049 | 0 | 0.960404 |
| TUBA4B | 0.02834 | 0.004049 | 0 | 0.960404 |
| C6 | 0.024291 | 0.004049 | 0.004049 | 0.960404 |
| CARD10 | 0.024291 | 0.004049 | 0.004049 | 0.960404 |
| CCNYL2 | 0.024291 | 0.004049 | 0.004049 | 0.960404 |
| CDK5RAP3 | 0.024291 | 0.004049 | 0.004049 | 0.960404 |
| F5 | 0.024291 | 0.004049 | 0.004049 | 0.960404 |
| MYOCD | 0.024291 | 0.004049 | 0.004049 | 0.960404 |
| PAPPA | 0.024291 | 0.004049 | 0.004049 | 0.960404 |
| PAPPA2 | 0.024291 | 0.004049 | 0.004049 | 0.960404 |
| TECTA | 0.024291 | 0.004049 | 0.004049 | 0.960404 |
| ACAN | 0.020243 | 0.004049 | 0.008097 | 0.960404 |
| AFF2 | 0.020243 | 0.004049 | 0.008097 | 0.960404 |
| COL18A1 | 0.020243 | 0.004049 | 0.008097 | 0.960404 |
| ENSG00000237452 | 0.020243 | 0.004049 | 0.008097 | 0.960404 |
| FBN1 | 0.020243 | 0.004049 | 0.008097 | 0.960404 |
| KSR2 | 0.020243 | 0.004049 | 0.008097 | 0.960404 |
| TBC1D1 | 0.020243 | 0.004049 | 0.008097 | 0.960404 |
| TNRC18 | 0.020243 | 0.004049 | 0.008097 | 0.960404 |
| VPS13A | 0.020243 | 0.004049 | 0.008097 | 0.960404 |
| FAM230A | 0.016194 | 0.004049 | 0.012146 | 0.960404 |
| SPTBN4 | 0.016194 | 0.004049 | 0.012146 | 0.960404 |
| STARD9 | 0.016194 | 0.004049 | 0.012146 | 0.960404 |
| TRIOBP | 0.016194 | 0.004049 | 0.012146 | 0.960404 |
| WNK1 | 0.016194 | 0.004049 | 0.012146 | 0.960404 |
| SCN1A | 0.012146 | 0.004049 | 0.016194 | 0.960404 |
| ARID2 | 0.024291 | 0.004049 | 0 | 1 |
| BSN | 0.024291 | 0.004049 | 0 | 1 |
| CACNA1F | 0.024291 | 0.004049 | 0 | 1 |
| CFTR | 0.024291 | 0.004049 | 0 | 1 |
| CPAMD8 | 0.024291 | 0.004049 | 0 | 1 |
| DNAJC13 | 0.024291 | 0.004049 | 0 | 1 |
| GPR123 | 0.024291 | 0.004049 | 0 | 1 |
| HTT | 0.024291 | 0.004049 | 0 | 1 |
| PREX2 | 0.024291 | 0.004049 | 0 | 1 |
| SHANK1 | 0.024291 | 0.004049 | 0 | 1 |
| ABCC12 | 0.020243 | 0.004049 | 0.004049 | 1 |
| ADAMTS20 | 0.020243 | 0.004049 | 0.004049 | 1 |
| AHNAK2 | 0.020243 | 0.004049 | 0.004049 | 1 |
| AMZ2 | 0.020243 | 0.004049 | 0.004049 | 1 |
| CAMTA1 | 0.020243 | 0.004049 | 0.004049 | 1 |
| DAPK1 | 0.020243 | 0.004049 | 0.004049 | 1 |
| DOCK3 | 0.020243 | 0.004049 | 0.004049 | 1 |
| FAM182B | 0.020243 | 0.004049 | 0.004049 | 1 |
| FMN2 | 0.020243 | 0.004049 | 0.004049 | 1 |
| FSIP2 | 0.020243 | 0.004049 | 0.004049 | 1 |
| GNAS | 0.020243 | 0.004049 | 0.004049 | 1 |
| GPR112 | 0.020243 | 0.004049 | 0.004049 | 1 |
| ITPR2 | 0.020243 | 0.004049 | 0.004049 | 1 |
| LILRB5 | 0.020243 | 0.004049 | 0.004049 | 1 |
| LINC00200 | 0.020243 | 0.004049 | 0.004049 | 1 |
| LOC285556 | 0.020243 | 0.004049 | 0.004049 | 1 |
| LRIG2 | 0.020243 | 0.004049 | 0.004049 | 1 |
| MYH4 | 0.020243 | 0.004049 | 0.004049 | 1 |
| PTHLH | 0.020243 | 0.004049 | 0.004049 | 1 |
| RIMBP2 | 0.020243 | 0.004049 | 0.004049 | 1 |
| RP1L1 | 0.020243 | 0.004049 | 0.004049 | 1 |
| SGK223 | 0.020243 | 0.004049 | 0.004049 | 1 |
| TCHHL1 | 0.020243 | 0.004049 | 0.004049 | 1 |
| THSD7A | 0.020243 | 0.004049 | 0.004049 | 1 |
| TSTD3 | 0.020243 | 0.004049 | 0.004049 | 1 |
| UNC79 | 0.020243 | 0.004049 | 0.004049 | 1 |
| VDAC1 | 0.020243 | 0.004049 | 0.004049 | 1 |
| ZFHX2 | 0.020243 | 0.004049 | 0.004049 | 1 |
| ZZZ3 | 0.020243 | 0.004049 | 0.004049 | 1 |
| ADCK5 | 0.016194 | 0.004049 | 0.008097 | 1 |
| ATM | 0.016194 | 0.004049 | 0.008097 | 1 |
| BCR | 0.016194 | 0.004049 | 0.008097 | 1 |
| CDH9 | 0.016194 | 0.004049 | 0.008097 | 1 |
| DNM1P46 | 0.016194 | 0.004049 | 0.008097 | 1 |
| DUSP27 | 0.016194 | 0.004049 | 0.008097 | 1 |
| DYNC2H1 | 0.016194 | 0.004049 | 0.008097 | 1 |
| LAMB1 | 0.016194 | 0.004049 | 0.008097 | 1 |
| MT-ND5 | 0.016194 | 0.004049 | 0.008097 | 1 |
| PLEKHG5 | 0.016194 | 0.004049 | 0.008097 | 1 |
| SHANK2 | 0.016194 | 0.004049 | 0.008097 | 1 |
| TESK2 | 0.016194 | 0.004049 | 0.008097 | 1 |
| THSD7B | 0.016194 | 0.004049 | 0.008097 | 1 |
| TNC | 0.016194 | 0.004049 | 0.008097 | 1 |
| TRIO | 0.016194 | 0.004049 | 0.008097 | 1 |
| TTC40 | 0.016194 | 0.004049 | 0.008097 | 1 |
| COL6A3 | 0.012146 | 0.004049 | 0.012146 | 1 |
| EPHA7 | 0.012146 | 0.004049 | 0.012146 | 1 |
| RP1 | 0.012146 | 0.004049 | 0.012146 | 1 |
| STAB2 | 0.012146 | 0.004049 | 0.012146 | 1 |
| TENM1 | 0.012146 | 0.004049 | 0.012146 | 1 |
| WDFY4 | 0.012146 | 0.004049 | 0.012146 | 1 |
| ANAPC2 | 0.008097 | 0.004049 | 0.016194 | 1 |
| DLGAP1 | 0.008097 | 0.004049 | 0.016194 | 1 |
| PTEN | 0.008097 | 0.004049 | 0.016194 | 1 |
| CUBN | 0.004049 | 0.004049 | 0.020243 | 1 |
| ANKRD18B | 0.016194 | 0.004049 | 0 | 1 |
| ATP2A3 | 0.016194 | 0.004049 | 0 | 1 |
| BMS1P8 | 0.016194 | 0.004049 | 0 | 1 |
| BRINP3 | 0.016194 | 0.004049 | 0 | 1 |
| CACNA1C | 0.016194 | 0.004049 | 0 | 1 |
| CCDC60 | 0.016194 | 0.004049 | 0 | 1 |
| CHD5 | 0.016194 | 0.004049 | 0 | 1 |
| CNTNAP4 | 0.016194 | 0.004049 | 0 | 1 |
| CR1 | 0.016194 | 0.004049 | 0 | 1 |
| CYP2C18 | 0.016194 | 0.004049 | 0 | 1 |
| EML5 | 0.016194 | 0.004049 | 0 | 1 |
| EMR1 | 0.016194 | 0.004049 | 0 | 1 |
| ENSG00000221664 | 0.016194 | 0.004049 | 0 | 1 |
| ENSG00000254420 | 0.016194 | 0.004049 | 0 | 1 |
| FER1L6 | 0.016194 | 0.004049 | 0 | 1 |
| HECTD4 | 0.016194 | 0.004049 | 0 | 1 |
| ITGA10 | 0.016194 | 0.004049 | 0 | 1 |
| KIAA1244 | 0.016194 | 0.004049 | 0 | 1 |
| LPHN2 | 0.016194 | 0.004049 | 0 | 1 |
| MAP3K5 | 0.016194 | 0.004049 | 0 | 1 |
| MSLNL | 0.016194 | 0.004049 | 0 | 1 |
| NBEA | 0.016194 | 0.004049 | 0 | 1 |
| OR2V2 | 0.016194 | 0.004049 | 0 | 1 |
| PCDH11Y | 0.016194 | 0.004049 | 0 | 1 |
| PHKA1 | 0.016194 | 0.004049 | 0 | 1 |
| SCN10A | 0.016194 | 0.004049 | 0 | 1 |
| SENP3 | 0.016194 | 0.004049 | 0 | 1 |
| SVEP1 | 0.016194 | 0.004049 | 0 | 1 |
| TNPO1 | 0.016194 | 0.004049 | 0 | 1 |
| UGT3A2 | 0.016194 | 0.004049 | 0 | 1 |
| UPF2 | 0.016194 | 0.004049 | 0 | 1 |
| ZNF234 | 0.016194 | 0.004049 | 0 | 1 |
| ABCA12 | 0.012146 | 0.004049 | 0.004049 | 1 |
| ANK2 | 0.012146 | 0.004049 | 0.004049 | 1 |
| ARHGEF28 | 0.012146 | 0.004049 | 0.004049 | 1 |
| BTBD11 | 0.012146 | 0.004049 | 0.004049 | 1 |
| C3 | 0.012146 | 0.004049 | 0.004049 | 1 |
| C6orf165 | 0.012146 | 0.004049 | 0.004049 | 1 |
| CAPN11 | 0.012146 | 0.004049 | 0.004049 | 1 |
| CHD9 | 0.012146 | 0.004049 | 0.004049 | 1 |
| CNTNAP3B | 0.012146 | 0.004049 | 0.004049 | 1 |
| COL4A3 | 0.012146 | 0.004049 | 0.004049 | 1 |
| CUL4B | 0.012146 | 0.004049 | 0.004049 | 1 |
| DENND2C | 0.012146 | 0.004049 | 0.004049 | 1 |
| ENSG00000188971 | 0.012146 | 0.004049 | 0.004049 | 1 |
| ENSG00000216113 | 0.012146 | 0.004049 | 0.004049 | 1 |
| ENSG00000221388 | 0.012146 | 0.004049 | 0.004049 | 1 |
| ENSG00000259241 | 0.012146 | 0.004049 | 0.004049 | 1 |
| EPHA8 | 0.012146 | 0.004049 | 0.004049 | 1 |
| FBLN2 | 0.012146 | 0.004049 | 0.004049 | 1 |
| GABRR1 | 0.012146 | 0.004049 | 0.004049 | 1 |
| GOLGA6L6 | 0.012146 | 0.004049 | 0.004049 | 1 |
| GON4L | 0.012146 | 0.004049 | 0.004049 | 1 |
| GRM3 | 0.012146 | 0.004049 | 0.004049 | 1 |
| HERC2P3 | 0.012146 | 0.004049 | 0.004049 | 1 |
| INF2 | 0.012146 | 0.004049 | 0.004049 | 1 |
| KCNH8 | 0.012146 | 0.004049 | 0.004049 | 1 |
| LILRB1 | 0.012146 | 0.004049 | 0.004049 | 1 |
| LINC00910 | 0.012146 | 0.004049 | 0.004049 | 1 |
| MAP1A | 0.012146 | 0.004049 | 0.004049 | 1 |
| MDGA2 | 0.012146 | 0.004049 | 0.004049 | 1 |
| MICAL3 | 0.012146 | 0.004049 | 0.004049 | 1 |
| MYH1 | 0.012146 | 0.004049 | 0.004049 | 1 |
| NBEAL1 | 0.012146 | 0.004049 | 0.004049 | 1 |
| NCOA1 | 0.012146 | 0.004049 | 0.004049 | 1 |
| NFAT5 | 0.012146 | 0.004049 | 0.004049 | 1 |
| OR4S1 | 0.012146 | 0.004049 | 0.004049 | 1 |
| PCDHA7 | 0.012146 | 0.004049 | 0.004049 | 1 |
| PLEC | 0.012146 | 0.004049 | 0.004049 | 1 |
| PLEKHA7 | 0.012146 | 0.004049 | 0.004049 | 1 |
| PLXNB1 | 0.012146 | 0.004049 | 0.004049 | 1 |
| PRG4 | 0.012146 | 0.004049 | 0.004049 | 1 |
| PRTG | 0.012146 | 0.004049 | 0.004049 | 1 |
| PTCHD2 | 0.012146 | 0.004049 | 0.004049 | 1 |
| PTGES | 0.012146 | 0.004049 | 0.004049 | 1 |
| RARB | 0.012146 | 0.004049 | 0.004049 | 1 |
| RASGRF1 | 0.012146 | 0.004049 | 0.004049 | 1 |
| REPIN1 | 0.012146 | 0.004049 | 0.004049 | 1 |
| RICTOR | 0.012146 | 0.004049 | 0.004049 | 1 |
| SCUBE1 | 0.012146 | 0.004049 | 0.004049 | 1 |
| SLC44A4 | 0.012146 | 0.004049 | 0.004049 | 1 |
| TM9SF2 | 0.012146 | 0.004049 | 0.004049 | 1 |
| TMPRSS15 | 0.012146 | 0.004049 | 0.004049 | 1 |
| TTLL5 | 0.012146 | 0.004049 | 0.004049 | 1 |
| TYRO3 | 0.012146 | 0.004049 | 0.004049 | 1 |
| YAF2 | 0.012146 | 0.004049 | 0.004049 | 1 |
| ADAMTSL3 | 0.008097 | 0.004049 | 0.008097 | 1 |
| AMZ1 | 0.008097 | 0.004049 | 0.008097 | 1 |
| CACNA1H | 0.008097 | 0.004049 | 0.008097 | 1 |
| CD163 | 0.008097 | 0.004049 | 0.008097 | 1 |
| CYP11B1 | 0.008097 | 0.004049 | 0.008097 | 1 |
| DIAPH1 | 0.008097 | 0.004049 | 0.008097 | 1 |
| ECE2 | 0.008097 | 0.004049 | 0.008097 | 1 |
| EHBP1 | 0.008097 | 0.004049 | 0.008097 | 1 |
| ENSG00000215953 | 0.008097 | 0.004049 | 0.008097 | 1 |
| FAT4 | 0.008097 | 0.004049 | 0.008097 | 1 |
| GREB1 | 0.008097 | 0.004049 | 0.008097 | 1 |
| HERC1 | 0.008097 | 0.004049 | 0.008097 | 1 |
| LINGO1 | 0.008097 | 0.004049 | 0.008097 | 1 |
| LOC101927209 | 0.008097 | 0.004049 | 0.008097 | 1 |
| LRRK1 | 0.008097 | 0.004049 | 0.008097 | 1 |
| MED12L | 0.008097 | 0.004049 | 0.008097 | 1 |
| MED14 | 0.008097 | 0.004049 | 0.008097 | 1 |
| NLRP9 | 0.008097 | 0.004049 | 0.008097 | 1 |
| OR5P2 | 0.008097 | 0.004049 | 0.008097 | 1 |
| PBX2P1 | 0.008097 | 0.004049 | 0.008097 | 1 |
| PTPRF | 0.008097 | 0.004049 | 0.008097 | 1 |
| SRRM2 | 0.008097 | 0.004049 | 0.008097 | 1 |
| SYNGAP1 | 0.008097 | 0.004049 | 0.008097 | 1 |
| TJP2 | 0.008097 | 0.004049 | 0.008097 | 1 |
| TMEM132D | 0.008097 | 0.004049 | 0.008097 | 1 |
| TSC2 | 0.008097 | 0.004049 | 0.008097 | 1 |
| TTC3 | 0.008097 | 0.004049 | 0.008097 | 1 |
| AP3D1 | 0.004049 | 0.004049 | 0.012146 | 1 |
| CDH18 | 0.004049 | 0.004049 | 0.012146 | 1 |
| CEL | 0.004049 | 0.004049 | 0.012146 | 1 |
| CRB1 | 0.004049 | 0.004049 | 0.012146 | 1 |
| DIS3L2 | 0.004049 | 0.004049 | 0.012146 | 1 |
| ENSG00000265002 | 0.004049 | 0.004049 | 0.012146 | 1 |
| FCHO1 | 0.004049 | 0.004049 | 0.012146 | 1 |
| FGFR1 | 0.004049 | 0.004049 | 0.012146 | 1 |
| HLA-DRB6 | 0.004049 | 0.004049 | 0.012146 | 1 |
| KIAA0040 | 0.004049 | 0.004049 | 0.012146 | 1 |
| VPS45 | 0 | 0.004049 | 0.016194 | 1 |
| BAI3 | 0.020243 | 0.004049 | 0 | 1 |
| CTBP2 | 0.020243 | 0.004049 | 0 | 1 |
| DOCK6 | 0.020243 | 0.004049 | 0 | 1 |
| GRIN3A | 0.020243 | 0.004049 | 0 | 1 |
| PCDHA8 | 0.020243 | 0.004049 | 0 | 1 |
| PHLDB1 | 0.020243 | 0.004049 | 0 | 1 |
| PPP1R12B | 0.020243 | 0.004049 | 0 | 1 |
| PTPRB | 0.020243 | 0.004049 | 0 | 1 |
| PTPRC | 0.020243 | 0.004049 | 0 | 1 |
| RAI1 | 0.020243 | 0.004049 | 0 | 1 |
| SCN11A | 0.020243 | 0.004049 | 0 | 1 |
| SFMBT2 | 0.020243 | 0.004049 | 0 | 1 |
| STAB1 | 0.020243 | 0.004049 | 0 | 1 |
| TACC2 | 0.020243 | 0.004049 | 0 | 1 |
| TAF1L | 0.020243 | 0.004049 | 0 | 1 |
| TMEM191C | 0.020243 | 0.004049 | 0 | 1 |
| UTP3 | 0.020243 | 0.004049 | 0 | 1 |
| XPNPEP1 | 0.020243 | 0.004049 | 0 | 1 |
| ANO7 | 0.016194 | 0.004049 | 0.004049 | 1 |
| ARHGAP29 | 0.016194 | 0.004049 | 0.004049 | 1 |
| CNTNAP2 | 0.016194 | 0.004049 | 0.004049 | 1 |
| COL12A1 | 0.016194 | 0.004049 | 0.004049 | 1 |
| COL6A5 | 0.016194 | 0.004049 | 0.004049 | 1 |
| DOCK8 | 0.016194 | 0.004049 | 0.004049 | 1 |
| FASN | 0.016194 | 0.004049 | 0.004049 | 1 |
| KMT2A | 0.016194 | 0.004049 | 0.004049 | 1 |
| KMT2D | 0.016194 | 0.004049 | 0.004049 | 1 |
| KRTAP4-5 | 0.016194 | 0.004049 | 0.004049 | 1 |
| LMO7 | 0.016194 | 0.004049 | 0.004049 | 1 |
| LOC150776 | 0.016194 | 0.004049 | 0.004049 | 1 |
| MPDZ | 0.016194 | 0.004049 | 0.004049 | 1 |
| MXRA5 | 0.016194 | 0.004049 | 0.004049 | 1 |
| OGDHL | 0.016194 | 0.004049 | 0.004049 | 1 |
| PCDHA6 | 0.016194 | 0.004049 | 0.004049 | 1 |
| PCDHB4 | 0.016194 | 0.004049 | 0.004049 | 1 |
| RBM5 | 0.016194 | 0.004049 | 0.004049 | 1 |
| RXRA | 0.016194 | 0.004049 | 0.004049 | 1 |
| SLIT2 | 0.016194 | 0.004049 | 0.004049 | 1 |
| SNRNP40 | 0.016194 | 0.004049 | 0.004049 | 1 |
| SPTB | 0.016194 | 0.004049 | 0.004049 | 1 |
| TRPM1 | 0.016194 | 0.004049 | 0.004049 | 1 |
| WASH6P | 0.016194 | 0.004049 | 0.004049 | 1 |
| ANKRD20A5P | 0.012146 | 0.004049 | 0.008097 | 1 |
| ARID1A | 0.012146 | 0.004049 | 0.008097 | 1 |
| COL4A1 | 0.012146 | 0.004049 | 0.008097 | 1 |
| DOCK7 | 0.012146 | 0.004049 | 0.008097 | 1 |
| ENSG00000216166 | 0.012146 | 0.004049 | 0.008097 | 1 |
| EOMES | 0.012146 | 0.004049 | 0.008097 | 1 |
| FLJ36000 | 0.012146 | 0.004049 | 0.008097 | 1 |
| KIAA2018 | 0.012146 | 0.004049 | 0.008097 | 1 |
| LAMA5 | 0.012146 | 0.004049 | 0.008097 | 1 |
| LOC440905 | 0.012146 | 0.004049 | 0.008097 | 1 |
| MT-RNR1 | 0.012146 | 0.004049 | 0.008097 | 1 |
| MYO7B | 0.012146 | 0.004049 | 0.008097 | 1 |
| NPAS3 | 0.012146 | 0.004049 | 0.008097 | 1 |
| OR2T8 | 0.012146 | 0.004049 | 0.008097 | 1 |
| OTOF | 0.012146 | 0.004049 | 0.008097 | 1 |
| PKD2 | 0.012146 | 0.004049 | 0.008097 | 1 |
| PLCZ1 | 0.012146 | 0.004049 | 0.008097 | 1 |
| PSG1 | 0.012146 | 0.004049 | 0.008097 | 1 |
| SDK1 | 0.012146 | 0.004049 | 0.008097 | 1 |
| SEC16A | 0.012146 | 0.004049 | 0.008097 | 1 |
| SLC22A8 | 0.012146 | 0.004049 | 0.008097 | 1 |
| TRPM2 | 0.012146 | 0.004049 | 0.008097 | 1 |
| USH1C | 0.012146 | 0.004049 | 0.008097 | 1 |
| ZNF610 | 0.012146 | 0.004049 | 0.008097 | 1 |
| ENSG00000221684 | 0.008097 | 0.004049 | 0.012146 | 1 |
| ESPNP | 0.008097 | 0.004049 | 0.012146 | 1 |
| HUWE1 | 0.008097 | 0.004049 | 0.012146 | 1 |
| LOC101927806 | 0.008097 | 0.004049 | 0.012146 | 1 |
| MYH2 | 0.008097 | 0.004049 | 0.012146 | 1 |
| PAXIP1 | 0.008097 | 0.004049 | 0.012146 | 1 |
| PRB3 | 0.008097 | 0.004049 | 0.012146 | 1 |
| ARFGEF1 | 0.004049 | 0.004049 | 0.016194 | 1 |
| LRRIQ3 | 0.004049 | 0.004049 | 0.016194 | 1 |
| ELFN2 | 0 | 0.004049 | 0.020243 | 1 |
| RB1 | 0.040486 | 0 | 0.068826 | 0.014431 |
| ENSG00000215933 | 0.048583 | 0 | 0.024291 | 0.06364 |
| USH2A | 0.048583 | 0 | 0.024291 | 0.06364 |
| MUC17 | 0.036437 | 0 | 0.016194 | 0.1445 |
| MGAM | 0.036437 | 0 | 0.008097 | 0.20173 |
| SSPO | 0.036437 | 0 | 0.008097 | 0.20173 |
| RNFT2 | 0.032389 | 0 | 0.012146 | 0.20173 |
| AHNAK | 0.024291 | 0 | 0.016194 | 0.238968 |
| MYH7 | 0.032389 | 0 | 0.004049 | 0.283773 |
| SEZ6L | 0.032389 | 0 | 0.004049 | 0.283773 |
| SPTA1 | 0.032389 | 0 | 0.004049 | 0.283773 |
| DUSP10 | 0.02834 | 0 | 0.008097 | 0.283773 |
| ALMS1 | 0.024291 | 0 | 0.012146 | 0.283773 |
| AOX2P | 0.020243 | 0 | 0.016194 | 0.283773 |
| FREM2 | 0.020243 | 0 | 0.016194 | 0.283773 |
| PCDH15 | 0.020243 | 0 | 0.016194 | 0.283773 |
| EYS | 0.02834 | 0 | 0.004049 | 0.338063 |
| NOTCH3 | 0.02834 | 0 | 0.004049 | 0.338063 |
| ZNF831 | 0.02834 | 0 | 0.004049 | 0.338063 |
| PAPD5 | 0.020243 | 0 | 0.012146 | 0.338063 |
| PLCE1 | 0.020243 | 0 | 0.012146 | 0.338063 |
| SCAND3 | 0.020243 | 0 | 0.012146 | 0.338063 |
| CTNNA2 | 0.016194 | 0 | 0.016194 | 0.338063 |
| KIAA0430 | 0.016194 | 0 | 0.016194 | 0.338063 |
| TLN2 | 0.016194 | 0 | 0.016194 | 0.338063 |
| AR | 0.012146 | 0 | 0.020243 | 0.338063 |
| MDN1 | 0.012146 | 0 | 0.020243 | 0.338063 |
| CECR2 | 0.02834 | 0 | 0 | 0.404455 |
| KALRN | 0.02834 | 0 | 0 | 0.404455 |
| NLRP4 | 0.02834 | 0 | 0 | 0.404455 |
| PRSS1 | 0.02834 | 0 | 0 | 0.404455 |
| ASPH | 0.024291 | 0 | 0.004049 | 0.404455 |
| BAZ2B | 0.024291 | 0 | 0.004049 | 0.404455 |
| CACNA1E | 0.024291 | 0 | 0.004049 | 0.404455 |
| DCDC1 | 0.024291 | 0 | 0.004049 | 0.404455 |
| FRMPD3 | 0.024291 | 0 | 0.004049 | 0.404455 |
| KIAA1109 | 0.024291 | 0 | 0.004049 | 0.404455 |
| KIAA1549 | 0.024291 | 0 | 0.004049 | 0.404455 |
| LAMA2 | 0.024291 | 0 | 0.004049 | 0.404455 |
| MYH9 | 0.024291 | 0 | 0.004049 | 0.404455 |
| MYO18B | 0.024291 | 0 | 0.004049 | 0.404455 |
| NFASC | 0.024291 | 0 | 0.004049 | 0.404455 |
| NOTCH4 | 0.024291 | 0 | 0.004049 | 0.404455 |
| SDK2 | 0.024291 | 0 | 0.004049 | 0.404455 |
| DROSHA | 0.020243 | 0 | 0.008097 | 0.404455 |
| FLG2 | 0.020243 | 0 | 0.008097 | 0.404455 |
| FRAS1 | 0.020243 | 0 | 0.008097 | 0.404455 |
| HNF1A | 0.020243 | 0 | 0.008097 | 0.404455 |
| MMP3 | 0.020243 | 0 | 0.008097 | 0.404455 |
| PTPRZ1 | 0.020243 | 0 | 0.008097 | 0.404455 |
| CDH26 | 0.016194 | 0 | 0.012146 | 0.404455 |
| COL22A1 | 0.016194 | 0 | 0.012146 | 0.404455 |
| GRIA1 | 0.016194 | 0 | 0.012146 | 0.404455 |
| MYCBP2 | 0.016194 | 0 | 0.012146 | 0.404455 |
| SACS | 0.016194 | 0 | 0.012146 | 0.404455 |
| WDR87 | 0.016194 | 0 | 0.012146 | 0.404455 |
| ZNF469 | 0.016194 | 0 | 0.012146 | 0.404455 |
| ASTN1 | 0.012146 | 0 | 0.016194 | 0.404455 |
| CASP5 | 0.012146 | 0 | 0.016194 | 0.404455 |
| CHL1 | 0.012146 | 0 | 0.016194 | 0.404455 |
| CNTNAP5 | 0.012146 | 0 | 0.016194 | 0.404455 |
| DLC1 | 0.012146 | 0 | 0.016194 | 0.404455 |
| EVPL | 0.008097 | 0 | 0.020243 | 0.404455 |
| PADI1 | 0.008097 | 0 | 0.020243 | 0.404455 |
| ASTN2 | 0.024291 | 0 | 0 | 0.486656 |
| ATP8B3 | 0.024291 | 0 | 0 | 0.486656 |
| C5orf42 | 0.024291 | 0 | 0 | 0.486656 |
| DSCAML1 | 0.024291 | 0 | 0 | 0.486656 |
| KNDC1 | 0.024291 | 0 | 0 | 0.486656 |
| LAMA3 | 0.024291 | 0 | 0 | 0.486656 |
| LRRC30 | 0.024291 | 0 | 0 | 0.486656 |
| MTTP | 0.024291 | 0 | 0 | 0.486656 |
| MUC19 | 0.024291 | 0 | 0 | 0.486656 |
| MYO16 | 0.024291 | 0 | 0 | 0.486656 |
| PDE1C | 0.024291 | 0 | 0 | 0.486656 |
| VPS13D | 0.024291 | 0 | 0 | 0.486656 |
| WNK2 | 0.024291 | 0 | 0 | 0.486656 |
| ZAK | 0.024291 | 0 | 0 | 0.486656 |
| ZMYM3 | 0.024291 | 0 | 0 | 0.486656 |
| CACNA2D3 | 0.020243 | 0 | 0.004049 | 0.486656 |
| DEAF1 | 0.020243 | 0 | 0.004049 | 0.486656 |
| GIMAP8 | 0.020243 | 0 | 0.004049 | 0.486656 |
| GVINP1 | 0.020243 | 0 | 0.004049 | 0.486656 |
| KIF1A | 0.020243 | 0 | 0.004049 | 0.486656 |
| LAMC1 | 0.020243 | 0 | 0.004049 | 0.486656 |
| MEFV | 0.020243 | 0 | 0.004049 | 0.486656 |
| MYO5C | 0.020243 | 0 | 0.004049 | 0.486656 |
| MYO7A | 0.020243 | 0 | 0.004049 | 0.486656 |
| PCDHA3 | 0.020243 | 0 | 0.004049 | 0.486656 |
| POTEM | 0.020243 | 0 | 0.004049 | 0.486656 |
| PPP1R3A | 0.020243 | 0 | 0.004049 | 0.486656 |
| RGPD4 | 0.020243 | 0 | 0.004049 | 0.486656 |
| SCN4A | 0.020243 | 0 | 0.004049 | 0.486656 |
| SKIDA1 | 0.020243 | 0 | 0.004049 | 0.486656 |
| SLC4A9 | 0.020243 | 0 | 0.004049 | 0.486656 |
| SLC8A3 | 0.020243 | 0 | 0.004049 | 0.486656 |
| SNHG14 | 0.020243 | 0 | 0.004049 | 0.486656 |
| TCOF1 | 0.020243 | 0 | 0.004049 | 0.486656 |
| TNR | 0.020243 | 0 | 0.004049 | 0.486656 |
| ZAP70 | 0.020243 | 0 | 0.004049 | 0.486656 |
| ZFHX3 | 0.020243 | 0 | 0.004049 | 0.486656 |
| ZNF536 | 0.020243 | 0 | 0.004049 | 0.486656 |
| ABCC3 | 0.016194 | 0 | 0.008097 | 0.486656 |
| ACACB | 0.016194 | 0 | 0.008097 | 0.486656 |
| ADAMTS18 | 0.016194 | 0 | 0.008097 | 0.486656 |
| ADAMTSL4 | 0.016194 | 0 | 0.008097 | 0.486656 |
| ARHGAP31 | 0.016194 | 0 | 0.008097 | 0.486656 |
| ASH1L | 0.016194 | 0 | 0.008097 | 0.486656 |
| CACNA1S | 0.016194 | 0 | 0.008097 | 0.486656 |
| CELSR1 | 0.016194 | 0 | 0.008097 | 0.486656 |
| CHD7 | 0.016194 | 0 | 0.008097 | 0.486656 |
| COL6A6 | 0.016194 | 0 | 0.008097 | 0.486656 |
| DENND4B | 0.016194 | 0 | 0.008097 | 0.486656 |
| ENSG00000216089 | 0.016194 | 0 | 0.008097 | 0.486656 |
| FAM230B | 0.016194 | 0 | 0.008097 | 0.486656 |
| GRID2 | 0.016194 | 0 | 0.008097 | 0.486656 |
| IGSF22 | 0.016194 | 0 | 0.008097 | 0.486656 |
| KCNT2 | 0.016194 | 0 | 0.008097 | 0.486656 |
| KHDRBS1 | 0.016194 | 0 | 0.008097 | 0.486656 |
| LAMC3 | 0.016194 | 0 | 0.008097 | 0.486656 |
| MMRN1 | 0.016194 | 0 | 0.008097 | 0.486656 |
| PRDM16 | 0.016194 | 0 | 0.008097 | 0.486656 |
| SMARCAL1 | 0.016194 | 0 | 0.008097 | 0.486656 |
| TENM3 | 0.016194 | 0 | 0.008097 | 0.486656 |
| TRPM5 | 0.016194 | 0 | 0.008097 | 0.486656 |
| WDR11 | 0.016194 | 0 | 0.008097 | 0.486656 |
| ENSG00000206195 | 0.012146 | 0 | 0.012146 | 0.486656 |
| KIAA1429 | 0.012146 | 0 | 0.012146 | 0.486656 |
| LRRC16A | 0.012146 | 0 | 0.012146 | 0.486656 |
| NOP56 | 0.012146 | 0 | 0.012146 | 0.486656 |
| POLE | 0.012146 | 0 | 0.012146 | 0.486656 |
| PYGM | 0.012146 | 0 | 0.012146 | 0.486656 |
| SNED1 | 0.012146 | 0 | 0.012146 | 0.486656 |
| TRPA1 | 0.012146 | 0 | 0.012146 | 0.486656 |
| UFL1 | 0.012146 | 0 | 0.012146 | 0.486656 |
| ABCA4 | 0.008097 | 0 | 0.016194 | 0.486656 |
| AP3B2 | 0.008097 | 0 | 0.016194 | 0.486656 |
| CCDC168 | 0.008097 | 0 | 0.016194 | 0.486656 |
| CENPE | 0.008097 | 0 | 0.016194 | 0.486656 |
| OTOP1 | 0.008097 | 0 | 0.016194 | 0.486656 |
| STAT2 | 0.008097 | 0 | 0.016194 | 0.486656 |
| URB2 | 0.008097 | 0 | 0.016194 | 0.486656 |
| ADAMTS3 | 0.020243 | 0 | 0 | 0.59019 |
| ADAMTS9 | 0.020243 | 0 | 0 | 0.59019 |
| AMBRA1 | 0.020243 | 0 | 0 | 0.59019 |
| C2CD5 | 0.020243 | 0 | 0 | 0.59019 |
| CADPS | 0.020243 | 0 | 0 | 0.59019 |
| CAPN13 | 0.020243 | 0 | 0 | 0.59019 |
| COBL | 0.020243 | 0 | 0 | 0.59019 |
| DAB1 | 0.020243 | 0 | 0 | 0.59019 |
| ENAM | 0.020243 | 0 | 0 | 0.59019 |
| FHOD3 | 0.020243 | 0 | 0 | 0.59019 |
| FLNC | 0.020243 | 0 | 0 | 0.59019 |
| FLT4 | 0.020243 | 0 | 0 | 0.59019 |
| FMO6P | 0.020243 | 0 | 0 | 0.59019 |
| HOOK1 | 0.020243 | 0 | 0 | 0.59019 |
| KDM2A | 0.020243 | 0 | 0 | 0.59019 |
| KIF19 | 0.020243 | 0 | 0 | 0.59019 |
| LCT | 0.020243 | 0 | 0 | 0.59019 |
| LRRC7 | 0.020243 | 0 | 0 | 0.59019 |
| MYH15 | 0.020243 | 0 | 0 | 0.59019 |
| MYH7B | 0.020243 | 0 | 0 | 0.59019 |
| MYT1L | 0.020243 | 0 | 0 | 0.59019 |
| NLRP2 | 0.020243 | 0 | 0 | 0.59019 |
| NOS3 | 0.020243 | 0 | 0 | 0.59019 |
| PCDHAC2 | 0.020243 | 0 | 0 | 0.59019 |
| PCSK5 | 0.020243 | 0 | 0 | 0.59019 |
| PXDNL | 0.020243 | 0 | 0 | 0.59019 |
| RCVRN | 0.020243 | 0 | 0 | 0.59019 |
| SLC17A6 | 0.020243 | 0 | 0 | 0.59019 |
| SLC4A3 | 0.020243 | 0 | 0 | 0.59019 |
| SNX14 | 0.020243 | 0 | 0 | 0.59019 |
| SOGA2 | 0.020243 | 0 | 0 | 0.59019 |
| ST18 | 0.020243 | 0 | 0 | 0.59019 |
| TENM4 | 0.020243 | 0 | 0 | 0.59019 |
| TMEM132B | 0.020243 | 0 | 0 | 0.59019 |
| UHRF1 | 0.020243 | 0 | 0 | 0.59019 |
| ZNF804A | 0.020243 | 0 | 0 | 0.59019 |
| ABCC8 | 0.016194 | 0 | 0.004049 | 0.59019 |
| ACSL6 | 0.016194 | 0 | 0.004049 | 0.59019 |
| ADAM28 | 0.016194 | 0 | 0.004049 | 0.59019 |
| ADAM6 | 0.016194 | 0 | 0.004049 | 0.59019 |
| ADNP2 | 0.016194 | 0 | 0.004049 | 0.59019 |
| AK9 | 0.016194 | 0 | 0.004049 | 0.59019 |
| ARHGAP21 | 0.016194 | 0 | 0.004049 | 0.59019 |
| ASTE1 | 0.016194 | 0 | 0.004049 | 0.59019 |
| ATP13A4 | 0.016194 | 0 | 0.004049 | 0.59019 |
| BCL11A | 0.016194 | 0 | 0.004049 | 0.59019 |
| C10orf68 | 0.016194 | 0 | 0.004049 | 0.59019 |
| C16orf96 | 0.016194 | 0 | 0.004049 | 0.59019 |
| C3orf20 | 0.016194 | 0 | 0.004049 | 0.59019 |
| C6orf132 | 0.016194 | 0 | 0.004049 | 0.59019 |
| CACNA1D | 0.016194 | 0 | 0.004049 | 0.59019 |
| CHD6 | 0.016194 | 0 | 0.004049 | 0.59019 |
| COPS4 | 0.016194 | 0 | 0.004049 | 0.59019 |
| DMBT1 | 0.016194 | 0 | 0.004049 | 0.59019 |
| DMXL1 | 0.016194 | 0 | 0.004049 | 0.59019 |
| DPYD | 0.016194 | 0 | 0.004049 | 0.59019 |
| FBXO5 | 0.016194 | 0 | 0.004049 | 0.59019 |
| FCRL3 | 0.016194 | 0 | 0.004049 | 0.59019 |
| FGD3 | 0.016194 | 0 | 0.004049 | 0.59019 |
| KDM5C | 0.016194 | 0 | 0.004049 | 0.59019 |
| KIAA0556 | 0.016194 | 0 | 0.004049 | 0.59019 |
| LOC100996870 | 0.016194 | 0 | 0.004049 | 0.59019 |
| LOC101927060 | 0.016194 | 0 | 0.004049 | 0.59019 |
| LOC101927648 | 0.016194 | 0 | 0.004049 | 0.59019 |
| MLIP | 0.016194 | 0 | 0.004049 | 0.59019 |
| MS4A14 | 0.016194 | 0 | 0.004049 | 0.59019 |
| MYT1 | 0.016194 | 0 | 0.004049 | 0.59019 |
| OR5L1 | 0.016194 | 0 | 0.004049 | 0.59019 |
| PCDHB13 | 0.016194 | 0 | 0.004049 | 0.59019 |
| PLG | 0.016194 | 0 | 0.004049 | 0.59019 |
| PTMAP5 | 0.016194 | 0 | 0.004049 | 0.59019 |
| PTPRT | 0.016194 | 0 | 0.004049 | 0.59019 |
| RBFOX1 | 0.016194 | 0 | 0.004049 | 0.59019 |
| RTEL1 | 0.016194 | 0 | 0.004049 | 0.59019 |
| SALL3 | 0.016194 | 0 | 0.004049 | 0.59019 |
| SEL1L2 | 0.016194 | 0 | 0.004049 | 0.59019 |
| SETD1B | 0.016194 | 0 | 0.004049 | 0.59019 |
| SLC7A9 | 0.016194 | 0 | 0.004049 | 0.59019 |
| SRPK2 | 0.016194 | 0 | 0.004049 | 0.59019 |
| SULF1 | 0.016194 | 0 | 0.004049 | 0.59019 |
| TAF1B | 0.016194 | 0 | 0.004049 | 0.59019 |
| TET1 | 0.016194 | 0 | 0.004049 | 0.59019 |
| TET3 | 0.016194 | 0 | 0.004049 | 0.59019 |
| TRPC3 | 0.016194 | 0 | 0.004049 | 0.59019 |
| TRPM6 | 0.016194 | 0 | 0.004049 | 0.59019 |
| TUBB8 | 0.016194 | 0 | 0.004049 | 0.59019 |
| ZIC4 | 0.016194 | 0 | 0.004049 | 0.59019 |
| ZNF335 | 0.016194 | 0 | 0.004049 | 0.59019 |
| ADARB2 | 0.012146 | 0 | 0.008097 | 0.59019 |
| ARHGAP5 | 0.012146 | 0 | 0.008097 | 0.59019 |
| ATRNL1 | 0.012146 | 0 | 0.008097 | 0.59019 |
| BCLAF1 | 0.012146 | 0 | 0.008097 | 0.59019 |
| BPTF | 0.012146 | 0 | 0.008097 | 0.59019 |
| BRD7 | 0.012146 | 0 | 0.008097 | 0.59019 |
| BRINP2 | 0.012146 | 0 | 0.008097 | 0.59019 |
| C9 | 0.012146 | 0 | 0.008097 | 0.59019 |
| CELSR3 | 0.012146 | 0 | 0.008097 | 0.59019 |
| CTTN | 0.012146 | 0 | 0.008097 | 0.59019 |
| DACT1 | 0.012146 | 0 | 0.008097 | 0.59019 |
| ENSG00000173213 | 0.012146 | 0 | 0.008097 | 0.59019 |
| ENSG00000266651 | 0.012146 | 0 | 0.008097 | 0.59019 |
| FRMD1 | 0.012146 | 0 | 0.008097 | 0.59019 |
| FSCB | 0.012146 | 0 | 0.008097 | 0.59019 |
| GOLGB1 | 0.012146 | 0 | 0.008097 | 0.59019 |
| GPR125 | 0.012146 | 0 | 0.008097 | 0.59019 |
| HDC | 0.012146 | 0 | 0.008097 | 0.59019 |
| IGF2BP1 | 0.012146 | 0 | 0.008097 | 0.59019 |
| IL2RB | 0.012146 | 0 | 0.008097 | 0.59019 |
| IQGAP1 | 0.012146 | 0 | 0.008097 | 0.59019 |
| LIG4 | 0.012146 | 0 | 0.008097 | 0.59019 |
| MBNL1 | 0.012146 | 0 | 0.008097 | 0.59019 |
| MED12 | 0.012146 | 0 | 0.008097 | 0.59019 |
| MTUS2 | 0.012146 | 0 | 0.008097 | 0.59019 |
| PAX4 | 0.012146 | 0 | 0.008097 | 0.59019 |
| PNPLA7 | 0.012146 | 0 | 0.008097 | 0.59019 |
| PSME4 | 0.012146 | 0 | 0.008097 | 0.59019 |
| SAMD9 | 0.012146 | 0 | 0.008097 | 0.59019 |
| SNORD3B-2 | 0.012146 | 0 | 0.008097 | 0.59019 |
| SPINK5 | 0.012146 | 0 | 0.008097 | 0.59019 |
| SPPL2B | 0.012146 | 0 | 0.008097 | 0.59019 |
| C14orf39 | 0.008097 | 0 | 0.012146 | 0.59019 |
| CFH | 0.008097 | 0 | 0.012146 | 0.59019 |
| ENSG00000265194 | 0.008097 | 0 | 0.012146 | 0.59019 |
| ENSG00000273433 | 0.008097 | 0 | 0.012146 | 0.59019 |
| HRCT1 | 0.008097 | 0 | 0.012146 | 0.59019 |
| KNTC1 | 0.008097 | 0 | 0.012146 | 0.59019 |
| LOC441666 | 0.008097 | 0 | 0.012146 | 0.59019 |
| LTBP3 | 0.008097 | 0 | 0.012146 | 0.59019 |
| LYST | 0.008097 | 0 | 0.012146 | 0.59019 |
| MLLT3 | 0.008097 | 0 | 0.012146 | 0.59019 |
| MYH11 | 0.008097 | 0 | 0.012146 | 0.59019 |
| OR8D1 | 0.008097 | 0 | 0.012146 | 0.59019 |
| PCNT | 0.008097 | 0 | 0.012146 | 0.59019 |
| PODXL | 0.008097 | 0 | 0.012146 | 0.59019 |
| PVRL2 | 0.008097 | 0 | 0.012146 | 0.59019 |
| ROS1 | 0.008097 | 0 | 0.012146 | 0.59019 |
| SYCP1 | 0.008097 | 0 | 0.012146 | 0.59019 |
| TRPM3 | 0.008097 | 0 | 0.012146 | 0.59019 |
| WDR66 | 0.008097 | 0 | 0.012146 | 0.59019 |
| LOC644794 | 0.004049 | 0 | 0.016194 | 0.59019 |
| MAN1B1 | 0.004049 | 0 | 0.016194 | 0.59019 |
| NEFM | 0.004049 | 0 | 0.016194 | 0.59019 |
