# Supplementary material for: Integrative Clustering Reveals a Novel Subtype of Soft Tissue Sarcoma With Poor Prognosis
Source: Front Genet. 2020 Feb 17;11:69. doi: 10.3389/fgene.2020.00069 (PMC7038822; doi:10.3389/fgene.2020.00069)
Supplement: Supplementary file 4 [file Table_3.docx]

Supplementary Table 3: Nodes of the ceRNA network

| Gene | Type | Cox_coefficient | P_value | Median_expression | HR_CL | logRank_P |
| --- | --- | --- | --- | --- | --- | --- |
| H19 | lnc | 0.00321 | 0.949 | 5.24 | [0.668-1.69] | 0.793 |
| DLGAP1-AS1 | lnc | -0.31 | 0.00805 | 2.37 | [0.449-1.14] | 0.159 |
| NEAT1 | lnc | -0.0668 | 0.443 | 8.18 | [0.567-1.44] | 0.666 |
| KCNQ1OT1 | lnc | 0.218 | 0.0126 | 2.25 | [1.31-3.32] | 0.00248 |
| hsa-miR-145-5p | mir | -0.0954 | 0.0648 | 9.86 | [0.539-1.37] | 0.523 |
| hsa-miR-149-5p | mir | 0.106 | 0.156 | 3.91 | [0.88-2.23] | 0.158 |
| hsa-miR-181b-5p | mir | 0.00147 | 0.989 | 8.66 | [0.489-1.24] | 0.294 |
| hsa-miR-29a-3p | mir | -0.146 | 0.12 | 11.3 | [0.438-1.11] | 0.131 |
| hsa-miR-29b-3p | mir | -0.0419 | 0.591 | 7.97 | [0.504-1.28] | 0.356 |
| hsa-miR-29c-3p | mir | -0.227 | 0.000863 | 7.99 | [0.326-0.833] | 0.00567 |
| hsa-miR-329-3p | mir | 0.0455 | 0.312 | 1.33 | [0.646-1.64] | 0.903 |
| hsa-miR-377-3p | mir | 0.00951 | 0.836 | 1.33 | [0.701-1.78] | 0.64 |
| TSPAN6 | pc | 0.387 | 0.000687 | 4.64 | [1.26-3.2] | 0.00354 |
| JARID2 | pc | 0.277 | 0.0199 | 4.09 | [1.15-2.92] | 0.0114 |
| CELSR3 | pc | 0.0386 | 0.556 | 2.65 | [0.659-1.67] | 0.841 |
| TSPAN9 | pc | 0.384 | 0.00586 | 6.37 | [0.898-2.28] | 0.132 |
| ELN | pc | -0.0781 | 0.109 | 7.21 | [0.377-0.958] | 0.0328 |
| CDON | pc | -0.067 | 0.341 | 4.06 | [0.565-1.44] | 0.661 |
| KIF26A | pc | 0.0144 | 0.814 | 2.96 | [0.504-1.28] | 0.357 |
| LRP6 | pc | -0.0976 | 0.411 | 5.06 | [0.618-1.57] | 0.945 |
| TRIB2 | pc | 0.161 | 0.0824 | 5.21 | [0.803-2.04] | 0.304 |
| FSCN1 | pc | 0.137 | 0.0737 | 7.95 | [1.08-2.75] | 0.0219 |
| PPP1R13B | pc | 0.138 | 0.261 | 4.15 | [0.656-1.66] | 0.855 |
| OTUB2 | pc | 0.227 | 0.0232 | 0.0608 | [0.855-2.17] | 0.192 |
| ZFHX4 | pc | 0.0441 | 0.324 | 4.05 | [0.96-2.44] | 0.0722 |
| TGFB2 | pc | 0.0466 | 0.528 | 3.73 | [0.884-2.24] | 0.154 |
| EPB41L4B | pc | 0.0113 | 0.839 | 1.17 | [0.859-2.19] | 0.182 |
| H2AFY2 | pc | -0.0242 | 0.735 | 3.96 | [0.589-1.49] | 0.79 |
| CBX7 | pc | -0.304 | 0.0013 | 4.83 | [0.433-1.1] | 0.118 |
| FNDC3A | pc | -0.00254 | 0.981 | 5.57 | [0.656-1.66] | 0.855 |
| ZNF423 | pc | -0.047 | 0.375 | 4.19 | [0.691-1.75] | 0.688 |
| CDK6 | pc | 0.165 | 0.0199 | 5.04 | [1.43-3.66] | 0.000517 |
| MEST | pc | 0.0808 | 0.0499 | 4.86 | [0.655-1.66] | 0.858 |
| PRKAG2 | pc | -0.155 | 0.0853 | 4.04 | [0.509-1.3] | 0.379 |
| PRUNE2 | pc | -0.0558 | 0.19 | 5.89 | [0.61-1.55] | 0.903 |
| MAP3K8 | pc | -0.23 | 0.0039 | 3.02 | [0.432-1.1] | 0.115 |
| MAP2K6 | pc | -0.0193 | 0.791 | 1.15 | [0.578-1.47] | 0.728 |
| USP46 | pc | 0.305 | 0.0129 | 3.91 | [0.934-2.38] | 0.0906 |
| DTX4 | pc | 0.0522 | 0.489 | 2.88 | [0.558-1.41] | 0.618 |
| BACH2 | pc | 0.0992 | 0.17 | 2.9 | [0.782-1.99] | 0.347 |
| DPYSL3 | pc | -0.149 | 0.0704 | 8.93 | [0.458-1.17] | 0.185 |
| RTKN | pc | 0.14 | 0.106 | 3.84 | [0.94-2.39] | 0.0879 |
| PLEK | pc | -0.143 | 0.0117 | 4.51 | [0.419-1.06] | 0.0919 |
| CD48 | pc | -0.154 | 0.00821 | 2.57 | [0.402-1.02] | 0.0622 |
| KDM5B | pc | 0.101 | 0.345 | 5.67 | [0.781-1.98] | 0.361 |
| CCND2 | pc | -0.00303 | 0.954 | 4.52 | [0.579-1.47] | 0.733 |
| DNMT3A | pc | 0.367 | 0.0142 | 5.07 | [1.11-2.82] | 0.0172 |
| CRISPLD1 | pc | 0.00252 | 0.958 | 3.73 | [0.708-1.8] | 0.613 |
| KIAA1549 | pc | 0.0686 | 0.264 | 2.1 | [0.706-1.79] | 0.624 |
| SOX9 | pc | 0.0771 | 0.0738 | 3.31 | [0.725-1.84] | 0.54 |
| HS3ST3B1 | pc | 0.0357 | 0.492 | 1.92 | [0.815-2.07] | 0.268 |
| ID1 | pc | 0.0297 | 0.694 | 3.8 | [0.705-1.79] | 0.626 |
| AMOT | pc | -0.0405 | 0.632 | 4.32 | [0.573-1.45] | 0.697 |
| RAB11FIP4 | pc | 0.0847 | 0.237 | 1.7 | [0.72-1.83] | 0.557 |
| TTLL7 | pc | -0.0646 | 0.286 | 3.85 | [0.548-1.39] | 0.565 |
| TET1 | pc | 0.193 | 0.0141 | 1.13 | [1.09-2.77] | 0.021 |
| FRAS1 | pc | 0.014 | 0.773 | 3.68 | [0.551-1.4] | 0.579 |
| RNF165 | pc | -0.0308 | 0.538 | 2.33 | [0.588-1.49] | 0.783 |
| VASH2 | pc | 0.0147 | 0.816 | 3.32 | [0.733-1.86] | 0.514 |
| EFNA3 | pc | 0.137 | 0.06 | 1.19 | [0.817-2.07] | 0.27 |
| LPP | pc | -0.154 | 0.0478 | 6.85 | [0.378-0.963] | 0.0333 |
| DAAM2 | pc | 0.00886 | 0.912 | 4.75 | [0.806-2.04] | 0.297 |
| SLC16A2 | pc | 0.212 | 0.047 | 4.69 | [0.746-1.89] | 0.47 |
| MPP7 | pc | 0.0136 | 0.749 | 1.45 | [0.683-1.73] | 0.721 |
| MBNL1 | pc | -0.271 | 0.0458 | 7.24 | [0.423-1.08] | 0.0973 |
| PPP1R9A | pc | 0.04 | 0.434 | 0.582 | [0.722-1.83] | 0.556 |
| CACHD1 | pc | 0.111 | 0.165 | 3.48 | [0.813-2.06] | 0.275 |
| NFIA | pc | -0.0427 | 0.654 | 5.93 | [0.543-1.38] | 0.546 |
| SLMAP | pc | -0.0764 | 0.267 | 5.58 | [0.478-1.21] | 0.251 |
| SPRY1 | pc | -0.00904 | 0.899 | 4.97 | [0.625-1.59] | 0.984 |
| CMYA5 | pc | -0.0308 | 0.523 | 1.95 | [0.532-1.35] | 0.483 |
| FZD6 | pc | 0.101 | 0.209 | 4.23 | [0.96-2.43] | 0.0761 |
| MAMDC2 | pc | -0.0908 | 0.07 | 3.71 | [0.507-1.29] | 0.369 |
| FRS2 | pc | 0.069 | 0.345 | 4.89 | [0.603-1.53] | 0.866 |
| TPP1 | pc | -0.295 | 0.0168 | 7.73 | [0.406-1.03] | 0.0684 |
| EFNA1 | pc | -0.00838 | 0.934 | 4.11 | [0.635-1.61] | 0.963 |
| ENC1 | pc | 0.0825 | 0.28 | 5.13 | [0.712-1.81] | 0.597 |
| NET1 | pc | 0.116 | 0.217 | 5.27 | [0.93-2.37] | 0.0939 |
| CBX2 | pc | 0.161 | 0.0812 | 3.65 | [0.677-1.72] | 0.753 |
| SOX11 | pc | 0.138 | 0.000664 | 1.37 | [1.02-2.59] | 0.0422 |
| MSC | pc | 0.0164 | 0.814 | 4.55 | [0.696-1.77] | 0.66 |
| ST6GALNAC3 | pc | 0.0586 | 0.561 | 2.29 | [0.627-1.59] | 0.994 |
| MITF | pc | -0.208 | 0.0119 | 4.07 | [0.332-0.847] | 0.00769 |
| SPRY4 | pc | 0.138 | 0.0623 | 4.73 | [0.807-2.05] | 0.294 |
| COL4A5 | pc | 0.0185 | 0.619 | 3.54 | [0.642-1.63] | 0.925 |
| TCF4 | pc | -0.0184 | 0.847 | 6.39 | [0.54-1.37] | 0.528 |
| NOL4L | pc | 0.095 | 0.506 | 4.44 | [0.641-1.63] | 0.929 |
| DNM3 | pc | -0.0373 | 0.692 | 2.31 | [0.594-1.51] | 0.817 |
| ZNF521 | pc | 0.0586 | 0.513 | 4.84 | [0.807-2.05] | 0.293 |
| PEG10 | pc | 0.153 | 0.00598 | 5.39 | [1.08-2.73] | 0.026 |
| ITGB3 | pc | -0.093 | 0.0657 | 3.3 | [0.499-1.27] | 0.335 |
